# Modeling the Cost-effectiveness of Esophageal Cancer Screening in China

**DOI:** 10.1186/s12962-020-00230-y

**Published:** 2020-09-10

**Authors:** Yuanyuan Li, Lingbin Du, Youqing Wang, Yuxuan Gu, Xuemei Zhen, Xiaoqian Hu, Xueshan Sun, Hengjin Dong

**Affiliations:** 1 Center for Health Policy Studies, School of Public Health, Zhejiang University School of Medicine, 866 Yuhangtang Rd., 310058 Hangzhou, Zhejiang China; 2 Department of Cancer Prevention, Institute of Cancer Research and Basic Medical Science of Chinese Academy of Sciences, Cancer Hospital of University of Chinese Academy of Sciences, Zhejiang Cancer Hospital, 38 Banshan Guangqiao Rd., 310022 Hangzhou, Zhejiang China

**Keywords:** Esophageal cancer, Markov model, Screening, Cost-effectiveness analysis

## Abstract

**Background:**

This study aimed to examine the cost-effectiveness of one-time standard endoscopic screening with Lugol’s iodine staining for esophageal cancer (EC) in China.

**Methods:**

A Markov decision analysis model with eleven states was built. Individuals aged 40 to 69 years were classified into six age groups in five-year intervals. Three different strategies were adopted for each cohort: (1) no screening; (2) one-time endoscopic screening with Lugol’s iodine staining with an annual follow-up for low-grade intraepithelial neoplasia (LGIN); and (3) one-time endoscopic screening with Lugol’s iodine staining without follow-up. Quality-adjusted life-years (QALYs) indicated the effectiveness of the model. The incremental cost-effectiveness ratio (ICER) was used as the evaluation indicator. Sensitivity analysis was performed to assess the robustness of the model.

**Results:**

One-time screening with follow-up was the undominated strategy for individuals aged 40–44 and 45–49 years, which saved USD 10,942.57 and USD 6611.73 per QALY gained *compared to nonscreening strategy*. For those aged 50–69 years, the nonscreening scenarios were undominated. One-time screening without follow-up was the extended dominated strategy. Compared to screening strategies without follow-up, all the screening strategies with follow-up were more cost-effective, with the ICER increasing from 299.57 USD/QALY for individuals aged 40–44 years to 1617.72 USD/QALY for individuals aged 65–69 years. Probabilistic sensitivity analysis (PSA) supported the results of the base case analysis.

**Conclusions:**

One-time EC screening with follow-up targeting individuals aged 40–49 years was the most cost-effective strategy.

## Background

Esophageal cancer (EC) is a malignant tumor that still ranks as the ninth most common cancer and the sixth leading cause of cancer-related death worldwide [1, 2]. There were 806,300 total cases, 472,500 new cases, and 436,000 deaths worldwide in 2017 [3]. EC incidence varies considerably according to geographical distribution, with over half of the worldwide incidence occurring in China [4]. The morbidity and mortality rates were 22.16 and 16.64 per 100,000 in 2013 in China [5]. Adenocarcinoma and esophageal squamous cell carcinoma (ESCC) are the major histological subtypes. Adenocarcinomas occur more frequently in developed countries, with an increasing trend in incidence, while ESCCs occur more frequently in less-developed regions [4]. ESCCs account for over 90% of all EC cases in China [6].

Several well-designed prospective preventive approaches have been developed to decrease the risk of EC; however, none have proven effective [7, 8]. Screening is a rapid, simple, and safe method that can be used to detect a disease in its early stage to reduce related risks and to treat the disease effectively. In the early 1980s, the WHO advocated secondary prevention strategies for early detection, diagnosis, and treatment. Cancer screening, as recommended by the WHO, is the major strategy for primary and secondary disease prevention [9]. More than 90% of EC patients have progressed to an advanced stage at the time of diagnosis due to asymptomatic characteristics in the early stage, with a 5-year survival rate of 15–20% [10]. Screening *can* detect approximately 90% of early-stage EC cases and thus significantly improve the 5-year survival rate *and also* identify patients in the precancerous stage, an unstable state in which the patient could return to health through lifestyle interventions.

Currently, there are no global EC screening guidelines. However, national EC screening was first performed in 2005 in China [11]. In 2010, the EC screening program was conducted in Zhejiang Province and performed strictly following the national guidelines for the early detection of cancer [12]. Although it was used to screen an entire population, not all the individuals were included due to budget restrictions. A random cluster sampling method was used to identify the participants. A total of 29,762 residents aged 40–69 years from seven counties in Zhejiang Province were screened between 2010 and 2017. In brief, the screening procedures were performed as follows. Eligible residents aged 40 to 69 years were invited to receive health education specific to EC. Then, prospective participants voluntarily participated in the screening and provided their signed informed consent. Afterwards, they were recommended to undergo a physical examination before they completed a baseline structural questionnaire related to the risks of developing EC. Individuals who met the following criteria were excluded from the screening tests: severe respiratory disease, serious heart failure, abnormal coagulation function, aortic aneurysm, acute stage of corrosive inflammation of the upper digestive tract, severe abdominal distension, iodine allergy history, ascites, and severe spinal deformity. Participants meeting the clinical screening criteria were then examined by standard endoscopy accompanied by Lugol’s iodine staining and indicative biopsy. All histological diagnoses were conducted by pathologists according to the AJCC cancer stage (seventh edition) [13]. Patients with clear histological diagnoses were recommended to undergo the following treatment. Patients with low-grade intraepithelial neoplasia (LGIN) were recommended to complete additional endoscopy screening in 1–3 years. Patients with intraductal carcinoma (IC) were recommended to undergo standard treatment (endoscopic submucosal dissection), while surgery was highly recommended for patients in the submucosal cancer (SM) stage. For patients with moderate-stage disease, surgery plus adjuvant chemoradiation was suggested, while patients with distant metastasis were treated with chemoradiation or treated for symptoms.

EC screening resulted in marked clinical benefits. First, the early diagnosis rates reached 70.59% to 98.1% [14]. In particular, a 10-year cohort study demonstrated decreased cumulative mortality (3.35% vs 5.05%) and cumulative incidence (4.17% vs 5.92%) in the screening population compared to the nonscreening population [15]. Moreover, the overall 5-year survival rate of patients with early EC reached 97.4–100%, and the disease-specific 5-year survival rate reached 100% [16–19]. However, the effectiveness of EC screening cannot be demonstrated without considering its economic value. An economic evaluation is useful, as it supports decision-making by providing an organized comparison of all available alternatives in terms of both their related costs and health outcomes [20]. Should the government launch a population-based EC screening program given the scarcity of medical resources and its relatively low incidence? Little is known about the cost and effectiveness of EC screening. This study aimed to compare the costs and effectiveness of one-time EC screening to identify the most cost-effective EC screening strategy and determine the age range for which screening should be performed in China.

## Methods

### Model structure

**Fig. 1:**
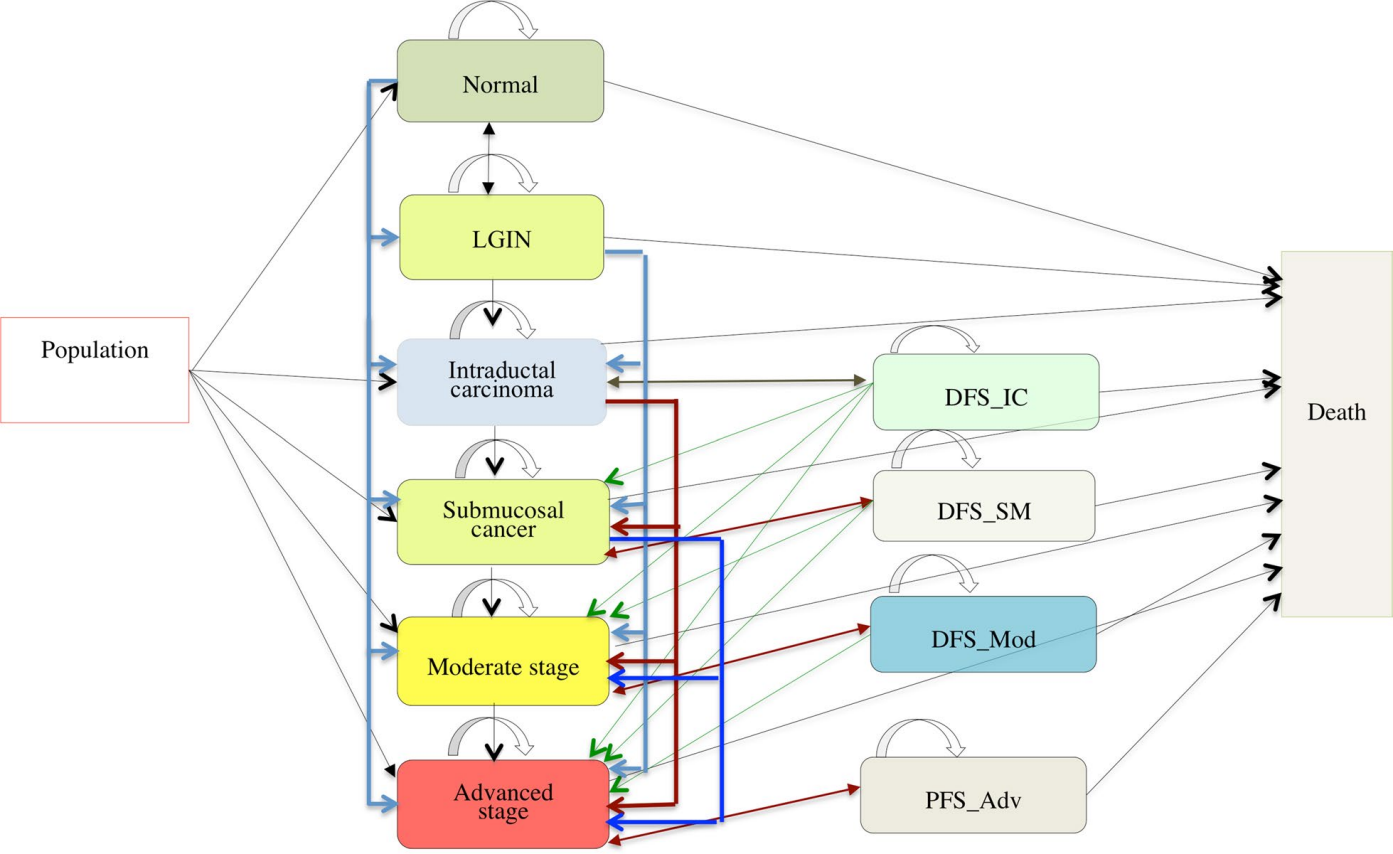
Markov model of EC progression with 11 health states

A decision analysis Markov model for EC with 11 health states was built with TreeAge Pro (2019). Only ESCC was modeled. The health states included normal, LGIN, IC, SM, moderate cancer stage (Mod), advanced cancer stage (Adv), disease-free survival state of IC (DFS_IC), disease-free survival state of SM (DFS_SM), disease-free survival state of moderate-stage cancer (DFS_Mod), progression-free survival state of advanced-stage cancer (PFS_Adv), and death. Here, in the nonscreening cohorts, “normal health” was assumed to be the state of non-EC, while in the screening cohorts, it was assumed to be the state of health without LGIN and EC. IC included high-grade intraepithelial neoplasia, while moderate stage included stage IB, stage II, and stage III. Stage IV was classified as an advanced cancer stage. Overall, both IC and SM constituted the early EC stage, while moderate and advanced stages were identified as the invasive EC stage. Figure 1 summarizes the state transition processes, with the arrows presenting the transitions between states.

Individuals aged 40 to 69 years were assumed to be participants and classified into six age groups separated by 5-year intervals (ages 40–44, 45–49, 50–54, 55–59, 60–64, and 65–69 years). Cohort simulation was performed until the cohort age reached 79 years or until hypothetical death. The model assumed a 1-year cycle length. A hypothetical cohort was generated with 100,000 individuals assigned to each age group. Three different strategies were assessed for each cohort. (1) Nonscreening, a strategy that assumes that all the individuals were not screened, and no endoscopic follow-up for LGIN was conducted. Patients were not diagnosed until clinical symptoms appeared, which is referred to as passive treatment. Over 90% of the patients in these cohorts had progressed into moderate and advanced stages. (2) Screening with an annual follow-up for LGIN, a strategy that assumes all the individuals undergo one-time standard endoscopic screening, which could identify LGIN and EC. Approximately 90% of EC patients were diagnosed at an early stage. (3) Screening without follow-up *for those with LGIN*, a strategy that assumes all the individuals underwent one-time standard endoscopic screening, which could identify LGIN and EC. Most patients with EC were in the early stage. The study assumed that all the patients diagnosed by the three strategies had the correct diagnosis and that the treatment strategies adhered to standardized treatment scenarios, including the standardized clinical follow-up recommended by the Guidelines for the Diagnosis and Treatment of EC 2018 in China.

### Effectiveness outcome

Quality-adjusted life-years (QALYs) represented the effectiveness of the model, and the incremental cost-effectiveness ratio (ICER) served as the economic evaluation indicator. The ICER indicates the additional cost per unit of additional effectiveness. The formula used to calculate the ICER was as follows: ICER = $\frac{{{\text{Cost}}_{\text{A}} - {\text{Cost}}_{\text{B}} }}{{{\text{QALY}}_{\text{A}} - {\text{QALY}}_{\text{B}} }}$. Cost-effectiveness analyses were used for comparisons between the competing strategies, including the “absolutely dominated strategy”, an option that had both more costs and less effectiveness; the “extended dominated strategy”, an option that was less costly and less effective than the alternative but had a higher ICER; and the “undominated best strategy”, an option that was cost-effective, with an ICER between 1 and 3 gross domestic products (GDPs) per capita based on the criterion recommended by the WHO [21]. Other outcomes assessed included costs, the cumulative incidence of EC, and mortality. Moreover, the willingness-to-pay (WTP) was set as three times the GDP per capita (USD 51,000) in 2017 in China.

### Cost and utility information

Costs were estimated from a social perspective. The estimation consisted of screening costs, treatment costs, transportation, and wage loss of patients and relatives due to hospital visits. Screening costs were calculated using the data of the screening program in Zhejiang Province, while treatment costs were extracted from electronic medical records at Zhejiang Cancer Hospital. The average annual costs of the disease-free survival states were computed with the following formula: C = ${\text{N}} \times \mathop \sum \nolimits_{1}^{\text{i}} {\text{i}} \times {\text{p}}_{\text{i}}$, where C is the annual costs of disease-free states, $\mathop \sum \nolimits_{1}^{\text{i}} {\text{i}} \times {\text{p}}_{\text{i}}$ is the average cost per visit, N is the annual average number of hospital visits, i is the number of physical examination items, and p is the price per item. N and i were suggested by the “Guidelines for the Diagnosis and Treatment of Esophageal Cancer 2018 in China”, and p was based on the price of medical services in provincial public hospitals in Zhejiang Province [22]. Transportation costs were computed using the second-class train or bus ticket price based on the distance between the visiting hospital and the patient’s home. Wage losses were calculated by multiplying 1 day’s income by the annual number of hospital visits (in days). We assumed that every patient had one accompanying relative. All costs were measured in the 2017 Chinese currency and were converted into US dollars using the purchasing power parity of 3.506 in 2017 [23]. All the items related to costs were assumed to inflate at the same inflation rate of 4.7% [24]. State-specific utilities were extracted from published papers [25–27]. A discount rate of 5% was used for both costs and effectiveness [28, 29]. Tables 1 and 2 display the state-specific costs and utilities, respectively.

**Table 1:** State-specific cost estimates for EC (USD)

State	Screening costs	Treatment-related costs	SA range	Distribution
Normal	60.3	0.0		
LGIN	60.3	149.5		
IC	60.3	17,561.9	±30 %	Gamma
SM	60.3	20,781.6		
Mod	60.3	25,217.4		
Adv	60.3	23,702.5		
DFS_IC	0.0	837.5		
DFS_SM	0.0	1580.0		
DFS_Mod	0.0	1580.0		
PFS_Adv	0.0	2873.0		
Death	0.0	0.0		

*SA* one-way sensitivity analysis

**Table 2:** State-specific utilities for EC

State	Mean	SD	SA range	Distribution	References
Normal	1.000	0.000	0.000–0.000	–	–
LGIN	0.941	0.089	0.753–1.000	Beta	[26]
IC	0.852	0.029	0.682–1.000	Beta	[25]
DFS_IC	0.940	0.100	0.752–1.000	Beta	[27]
SM	0.693	0.310	0.554–0.832	Beta	[25]
DFS_SM	0.870	0.150	0.696–1.000	Beta	[27]
Mod	0.780	0.140	0.624–0.936	Beta	[27]
DFS_Mod	0.810	0.170	0.648–0.972	Beta	[27]
Adv	0.720	0.180	0.576–0.864	Beta	[27]
PFS_Adv	0.740	0.190	0.592–0.888	Beta	[27]
Death	0.000	0.000	0.000–0.000	–	–

*SA* one-way sensitivity analysis

### Data analysis

#### Probabilities in the initial cohort

The probabilities for EC states in the initial nonscreening cohorts were calculated according to the 2012 age-specific incidence of EC in Zhejiang Province multiplied by the stage distribution at the time of diagnosis that was obtained from the hospital-based retrospective study [30]. The probabilities for EC states in the screening cohorts were computed by the age-specific EC detection rate multiplied by the stage distribution that was distinguished by screening. The initial probabilities of LGIN in the screening cohorts were the age-specific LGIN detection rate obtained from the screening program, while the initial probabilities in the nonscreening cohorts, were assumed to be zero since LGIN would not be diagnosed without screening. The probabilities for the normal state were one minus the sum of the probabilities for the other states for both scenarios. Table 3 displays the age-specific incidence and detection rate of EC and the age-specific detection rate of LGIN, while Table 4 displays the stage distributions for both scenarios.

**Table 3:** Age-specific EC incidence, detection rate and mortality (per 100,000)

Age	EC annual incidence	EC annual incidence for LGIN	EC detection rate	LGIN detection rate	EC-related mortality	All-cause mortality
40–44	1.36	4.96	16.92	130.56	0.68	122.41
45–49	4.90	17.68	70.65	545.00	2.19	184.60
50–54	9.93	34.95	193.45	1492.33	7.57	343.55
55–59	25.41	84.00	518.59	4000.54	14.87	475.18
60–64	35.48	108.02	976.17	7530.48	22.03	738.42
65–69	46.25	118.02	2072.48	15,987.72	33.08	1262.28
70–74	63.97	163.22	–	–	53.42	2380.26
75–79	64.45	164.36	–	–	65.73	4094.67

One-way sensitivity analysis values ± 20%; beta distribution was assumed for probability sensitivity analysis

**Table 4:** Stage distributions of EC under the screening and nonscreening scenarios

Stage	Nonscreening (%)		Screening (%)		Distribution
	Proportion	SA range	Proportion	SA range	
IC	3.65	2.92–4.38	88.24	70.59–100.00	Dirichlet
SM	4.93	3.94–5.92	2.52	2.02–3.02	
Mod	66.06	52.85–79.27	6.72	5.38–8.06	
Adv	25.36	20.29–30.43	2.52	2.02–3.02	

*SA* one-way sensitivity analysis

#### Transition probabilities between Markov states

The annual incidence of EC was used to calculate the probabilities of transition from the normal state to the EC state for the nonscreening cohorts, while in the screening cohorts, the adjusted annual incidence of EC was used to compute the probabilities of transition. The formula used was I_a_ = I_p_ × RR, where I_p_ is the annual incidence of EC, I_a_ is the adjusted annual incidence of EC, and RR is the annual incidence probability ratio that was computed with the formula used for the conversion between rate and probability using the cumulative incidence of ESCC in the screening group versus the nonscreening group [15, 31]. Moreover, the stage distribution at the time of diagnosis by passive treatment was used to identify EC states in both scenarios.

The probabilities of transferring from the LGIN state to the EC state were computed by the annual incidence of EC among the patients with LGIN (Table 3) multiplied by the EC stage distribution. The major difference was that the EC stage distribution was obtained from the results of screening programs for cohort with follow-up, while it was obtained from the results of passive treatment strategies without follow-up. In addition, the model assumed that a proportion of LGIN patients transferred to a normal state for strategies with follow-up [32–38], while no individuals transferred from an LGIN state to a normal state for strategies without follow-up. The incidence of EC among the LGIN patients was the adjusted incidence according to the risk ratio of the incidence of EC among LGIN patients compared to that among healthy subjects combined with the detected proportion of LGIN patients during screening. The risk ratio, which was summarized from published papers, was 3.66 [39–42]. No individuals were transferred from the LGIN state to the EC state under the nonscreening scenario since LGIN was not diagnosed due to asymptomatic characteristics without the implementation of screening. Other probabilities of transitioning between EC states were collected from various reports (Table 5).

**Table 5:** Other parameters incorporated in the model

Parameter	Input	SA range	Distribution	References
Transition probability				
Normal to LGIN	0.0000	–	–	–
LGIN to normal	0.1427	0.1142–0.1712	Beta	[32–38]
IC to DFS_IC	0.9363-d_nor	0.7490–0.9363	Beta	[17]
IC progress	0.0534	0.0427–0.0641	Beta	[17, 47–49]
SM proportion	0.2143	0.1714–0.2572	Dirichlet	Screening
Mod proportion	0.5714	0.4571–0.6857	Dirichlet	
Adv proportion	0.2143	0.1714–0.2572	Dirichlet	
DFS_IC to IC	0.0069	0.0055–0.0083	Beta	[50]
DFS_IC progress	0.0268	0.0214–0.0322	Beta	[51]
SM proportion	0.5556	0.4445–0.6667	Dirichlet	[51]
Mod proportion	0.4444	0.3555–0.5333	Dirichlet	[51]
Adv proportion	0.0000	0.0000–0.0000	Dirichlet	[51]
SM to DFS_SM	0.9051-d_nor	0.7241–1.0000	Beta	[52]
SM progress	0.1386	0.1109–0.1663	Beta	[53, 54]
Mod proportion	0.7562	0.6050–0.9074	Beta	[48, 55–61]
Adv proportion	0.2438	0.1950–0.2926	Beta	[48, 55–61]
DFS_SM to SM	0.0393	0.0314–0.0472	Beta	[62]
DFS_SM progress	0.0883	0.0706–0.1060	Beta	[53]
Mod proportion	0.7562	0.6050–0.9074	Beta	[48, 55–61]
Adv proportion	0.2438	0.1950–0.2926	Beta	[48, 55–61]
Mod to DFS_Mod	0.5930-d_nor	0.4744–0.7116	Beta	[63]
Mod to Adv	0.0317	0.0254–0.0380	Beta	[64]
DFS_Mod to Mod	0.0425	0.0340–0.0510	Dirichlet	[64]
DFS_Mod to Adv	0.0097	0.0078–0.0116	Dirichlet	[64]
Adv to PFS	0.1967-d_nor	0.1574–0.2360	Dirichlet	[65]
PFS progress	0.7002	0.5602–0.8402	Dirichlet	[66]
RR	0.7000	0.5600–0.8400	Lognormal	[15]
EC state-specific death probability (age ≤ 65 years)				
SM	0.0994	0.0795–0.1193	Beta	[67]
DFS_SM	0.0633	0.0506–0.0760	Beta	[67]
Mod	0.2988	0.2390–0.3586	Beta	[54]
DFS_Mod	0.1902	0.1522–0.2282	Beta	[54]
Adv	0.4613	0.3690–0.5536	Beta	[66, 68]
PFS	0.4303	0.3442–0.5164	Beta	[66, 68]
Risk ratios of EC death probability among patients aged more than 65 years compared to patients aged less than 65 years				
RR_SM/RR_DFS_SM	1.30	1.20–1.50	Lognormal	[69]
RR_Mod/RR_DFS_Mod	1.20	1.10–1.30	Lognormal	[69]
RR_Adv/RR_PFS_Adv	1.16	1.10–1.20	Lognormal	[69]

*SA* one-way sensitivity analysis, *d_nor* annual death probability for health individuals, which was defined as the difference between all-cause mortality and EC-related mortality

The age-specific annual death probabilities for the normal state were defined as the difference between all-cause mortality and EC-related mortality. All-cause mortality was obtained from the sixth population service survey, while EC-related mortality was obtained from data on age-specific EC mortality in Zhejiang Province in 2012 [30, 43]. LGIN was considered a precancerous lesion. The EC-specific 5-year survival rate was 100% for patients with IC and DFS_IC; therefore, people with LGIN or IC were not likely to die from EC. Consequently, the mortality rates for patients with LGIN, IC, and DFS_IC were assumed to be the same as those for patients in the normal state (Table 3). Death probabilities associated with SM and invasive cancer were identified from published papers, while the mortality risk was adjusted according to age (Table 3).

Moreover, a cycle length of 1 year was chosen; therefore, all the probabilities of transitions between states are presented as one-year probabilities. Given the different follow-up periods in the various data sources, we used two-step calculations. First, we converted the t-year follow-up probabilities into one-year rates. Then, we calculated one-year probabilities using one-year rates. The following formula was used to calculate the relationship between rate and probability. r = $- \frac{{{ \ln }\left( {1 - {\text{p}}} \right)}}{\text{t}}$; p = 1 − exp (− rt), where r indicates the rate, p indicates the probability, and t indicates the years of follow-up [31].

#### Sensitivity analysis

Sensitivity analyses for cost-effective screening strategies were performed. Probabilistic sensitivity analysis (PSA) permits the joint uncertainty across all the parameters in the model to be assessed at the same time, which involves sampling model parameter values from the distribution imposed on variables in the model. Initial cohort probabilities and death probabilities are assumed to be beta distributions, while the discount rate and inflation rate are considered triangular distributions. A gamma distribution was set for costs, and beta and Dirichlet distributions were set for transition probabilities. In addition, one-way sensitivity analyses were simulated because the impact of each parameter estimate varied independently and singly on the model results. The range of each parameter was assumed to be simply a “plausible” range. The following assumption was made. Initial probabilities, state transition probabilities, risk ratios, and health utility varied by ± 20% of the base case value, while costs varied by ± 30% of the base case value. Moreover, 0–8% was simulated for the discount rate, and 3.2–6.2% was used for the inflation rate.

## Results

### Simulated cumulative EC incidence and mortality

Table 6 provides the details of the simulation. Compared to nonscreening, screening reduced the simulated cumulative EC incidence across all age groups. Strategies with follow-up reduced cumulative EC incidence more than strategies without follow-up. The reduction in cumulative EC incidence decreased with increasing screening age. Cumulative EC incidence was reduced by 29.9% to 18.9% and 29.6% to 2.0% among individuals aged 40 to 69 years in screening strategies with and without follow-up, respectively. Compared to screening strategies without follow-up, strategies with follow-up reduced cumulative EC incidence across all age groups. The reduction in cumulative EC incidence increased with increasing screening age. Cumulative EC incidence was reduced by 0.4% to 17.2% in individuals aged 40 to 69 years.

**Table 6:** Summary of simulated cumulative EC incidence and mortality among different screening strategies

Age	Strategy	CI (per 100,000)	CM (per 100,000)	RCI (%)		RCM (%)	
				Scr* vs No_Scr	Scr_fol vs Scr_nfol	Scr* vs No_Scr	Scr_fol vs Scr_nfol
40–44	Non_scr	1010.62	776.76	–	–	–	–
	Scr_nfol	711.21	555.45	− 29.63	–	− 28.49	–
	Scr_fol	708.61	553.32	− 29.88	− 0.37	− 28.77	− 0.38
45–49	Non_scr	1010.04	778.32	–	–	–	–
	Scr_nfol	719.08	587.97	− 28.81	–	− 24.46	–
	Scr_fol	708.81	579.22	− 29.82	− 1.43	− 25.58	− 1.49
50–54	Non_scr	994.97	766.49	–	–	–	–
	Scr_nfol	726.70	642.84	− 26.96	–	− 16.13	–
	Scr_fol	701.17	619.77	− 29.53	− 3.51	− 19.14	− 3.59
55–59	Non_scr	962.42	746.15	–	–	–	–
	Scr_nfol	748.82	767.25	− 22.19	–	2.83	–
	Scr_fol	691.03	709.45	− 28.20	− 7.72	− 4.92	− 7.53
60–64	Non_scr	857.84	649.89	–	–	–	–
	Scr_nfol	720.35	829.78	− 16.03	–	27.68	–
	Scr_fol	637.03	738.52	− 25.74	− 11.57	13.64	− 11.00
65–69	Non_scr	710.26	512.38	–	–	–	–
	Scr_nfol	696.12	952.77	− 1.99	–	85.95	–
	Scr_fol	576.12	806.34	− 18.89	− 17.24	57.37	− 15.37

*Scr_fol* screening with follow-up, *Scr_nfol* screening without follow-up, *Non_scr* nonscreening, Scr* screening with or without follow-up, *CI* cumulative EC incidence, *CM* cumulative EC mortality, *RCI* reduction in cumulative incidence compared to an alternative strategy, *RCM* reduction in cumulative mortality compared to an alternative strategy

Compared to nonscreening, screening with follow-up for individuals aged 40–59 years resulted in a significant decrease in cumulative EC mortality but an obvious increase in cumulative EC mortality for individuals aged 60–69 years. The reduction in cumulative EC mortality varied from 28.8 to 4.9% among individuals between 40 and 59 years of age. Strategies without follow-up resulted in a considerable decrease in cumulative EC mortality for individuals 40–54 years of age but an obvious increase for individuals 55–69 years of age. The reduction decreased from 28.5 to 16.1% in individuals aged 40–44 years to individuals aged 50–54 years. Compared to strategies without follow-up, all the strategies with follow-up resulted in a significant decrease in cumulative EC mortality. The reduction increased by 0.4% to 15.4% in individuals aged 40–44 years to individuals aged 65–69 years.

### ICERs

The detailed results of the base case analyses are displayed in Table 7. Screening with follow-up was the undominated strategy, with USD 10,942.57 and USD 6611.73 saved per QALY gained for individuals aged 40–44 and 45–49 years, respectively, *compared to nonscreening*. For individuals aged 50–69 years, nonscreening was the undominated strategy. Screening without follow-up was the extended dominated strategy. Compared to screening strategies without follow-up, all the strategies *using* follow-up were cost-effective, with the ICER increasing from 299.57 USD/QALY for individuals aged 40–44 years to 1617.72 USD/QALY for individuals aged 65–69 years.

**Table 7:** Summary of cost-effectiveness analyses among different EC screening strategies

Age	Strategy	Costs* (USD: million)	QALYs (1000 years)	ICER	ICER
				Scr* vs No_Scr	Scr_fol vs Scr_nfol
40–44	Non_scr	40.02	1655.24	–	–
	Scr_nfol	35.14	1655.59	ED	–
	Scr_fol	35.17	1655.69	− 10,942.57	299.57
45–49	Non_scr	40.63	1557.40	–	–
	Scr_nfol	38.65	1557.31	ED	–
	Scr_fol	38.78	1557.68	− 6611.73	359.67
50–54	Non_scr	40.52	1437.30	–	–
	Scr_nfol	44.85	1436.15	ED	–
	Scr_fol	45.29	1437.07	AD	471.63
55–59	Non_scr	39.83	1295.22	–	–
	Scr_nfol	58.84	1291.50	ED	–
	Scr_fol	60.30	1293.66	AD	675.76
60–64	Non_scr	35.20	1120.73	–	–
	Scr_nfol	70.82	1114.21	ED	–
	Scr_fol	74.17	1117.47	AD	1026.61
65–69	Non_scr	28.49	908.42	–	–
	Scr_nfol	95.20	896.88	ED	–
	Scr_fol	103.19	901.82	AD	1617.72

*Scr_fol* screening with follow-up, *Scr_nfol* screening without follow-up, *Non_scr* nonscreening, *ICER* incremental cost-effectiveness ratio, Scr* screening with or without follow-up, *AD* absolutely dominated strategy, which was the option that had both more costs and less effectiveness, *ED* extended dominated strategy, which was the option that was less costly and less effective than the alternative but had a higher ICER

Costs* stage and state-specific costs are displayed in eTable 1 and eTable 2

### Sensitivity analyses

**Fig. 2:**
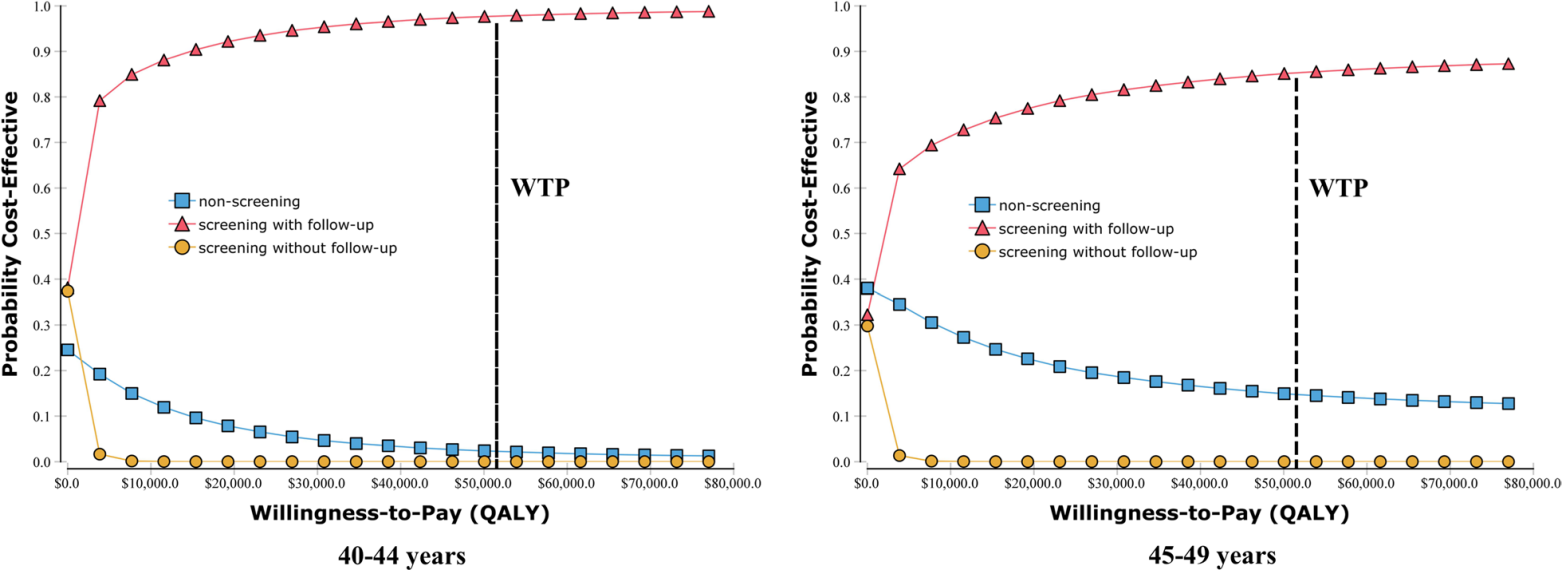
Cost-effectiveness acceptability curve for EC screening

**Fig. 3:**
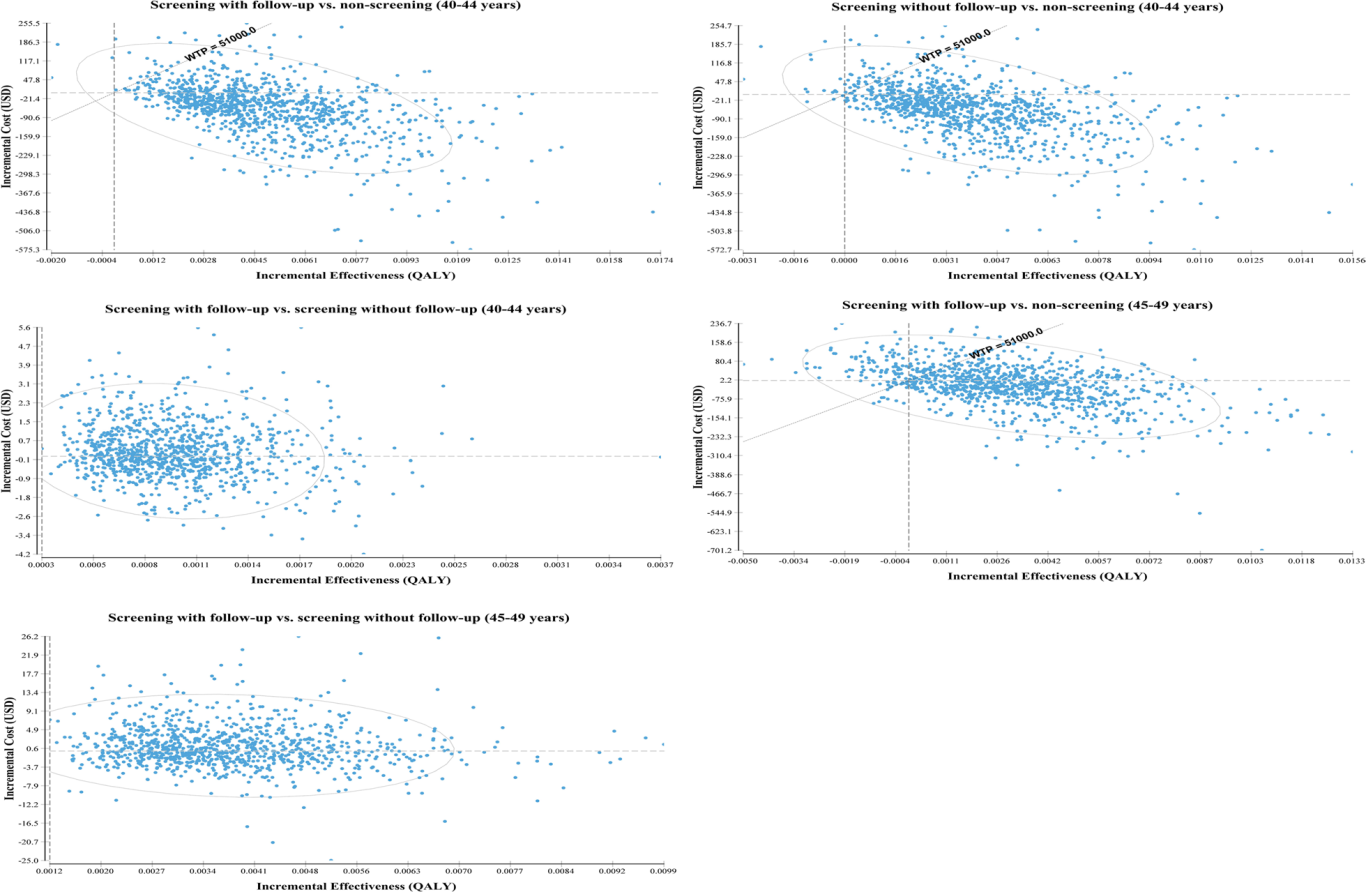
Incremental cost-effective ratios plotted on a cost-effectiveness plane among different screening strategies

**Fig. 4:**
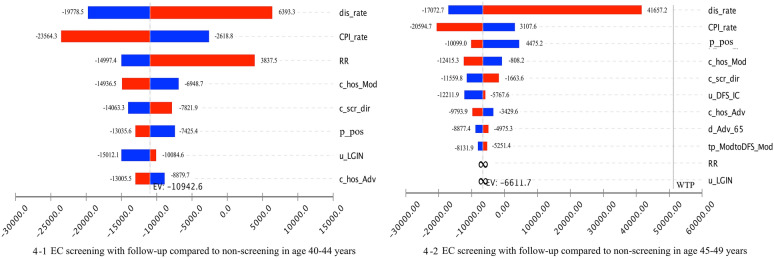
Tornado diagram assessing the effect of the uncertainty of a single parameter on the ICER (USD/QALY)

We ran 100,000 bootstrap interactions in the PSA. The primary results of the PSA are shown in Fig. 2. The findings revealed that considering a WTP of three times the GDP per QALY, screening with follow-up was the preferred strategy. The screening strategies without follow-up were dominated by the screening strategies with follow-up, with less costs and less QALYs than the nonscreening strategies. Figure 3 presents the differences in costs and QALYs per person between different screening strategies, along with a confidence ellipse that was computed using joint probability density. The confidence ellipse encompassed 95% of observations given the assumed WTP. More than 84% of the incremental costs and effectiveness scatters of screening with follow-up over nonscreening are plotted in the south-east & north-west quadrants, while all of the observations of screening with follow-up over screening without follow-up are plotted in the south-east and north-west quadrants. The outcome of the one-way sensitivity analysis is reported as a “tornado diagram” (Fig. 4), and only the parameters that accounted for 99% of the cumulative risk related to the ICER are displayed. The calculated ICER in the tornado diagram compared the screening with follow-up strategy to the nonscreening strategy. Figure 4 shows that the discount rate had a large impact on the ICER for individuals aged 40–44 years. An increase in the discount rate could reduce the cost-effectiveness of screening. However, even when the discount rate increased to 8%, the ICER was less than the WTP. For individuals aged 45–49 years, increasing the base case value of the RR to 0.84 and u_LGIN to 1 could make screening become the absolutely dominated strategy.

## Discussion

This study was the first to examine the cost-effectiveness of population-based EC screening. A Markov decision analysis model was conducted based on the natural history of EC to compare the ICER of screening with or without follow-up to nonscreening. The model simulated the postoperative states, aligning well with the natural progression of EC, by assessing disease-free survival and progression-free survival for patients with early-, moderate-, and late-stage EC. Age was highly correlated with disease incidence and mortality. Hence, in this study, cohort simulation with six age groups separated by five-year intervals was performed until the cohort age reached 79 years or hypothetical death. Each age bracket was compared to its nonscreening group, which improved the comparability among strategies within age groups. In addition, the study assumed age-specific epidemiological assumptions, including age-specific initial probabilities, mortality, and transition probabilities. Furthermore, we calculated not only the direct medical costs but also the indirect costs using the human capital method.

Based on the criterion for cost-effectiveness recommended by the WHO [21], this study found that one-time EC screening targeting patients aged 40–49 years showed long-term cost-effectiveness. Though the data sources of EC incidence, detection, and cancer stage distributions derived from Zhejiang Province were used to calibrate the model parameters, the incidence and mortality in the screening areas in Zhejiang Province were quite comparable to those in China. Although Zhejiang-based costs were chosen, according to the one-way sensitivity analysis, a decrease or an increase by 30% of the costs obtained by screening with follow-up targeting patients with moderate- and advanced-stage EC aged 40–44 and 45–49 years still saved costs per QALY gained. Additionally, PSA confirmed that joint uncertainty across these parameters had no impact on the cost-effectiveness of screening targeting individuals in the same age group. Consequently, the results of this study suggest the cost-effectiveness of national one-time EC screening targeting individuals aged 40–49 years. Moreover, this screening should be performed with endoscopic follow-up, which enhances the benefits of screening for relatively small additive costs. Follow-up observation plays a key role in reducing EC mortality and morbidity. It not only promotes the transition of the LGIN state to a normal state through lifestyle interventions but also detects early-stage cancer and allows it to be treated early. Adenocarcinoma is the predominant histologic subtype in developed countries and shows an increasing trend in incidence. Our model simulated only the natural history of ESCC, and we cannot generalize the cost-effectiveness of ESCC screening to the whole world due to different natural histories and cost data. The cost-effectiveness of adenocarcinoma should be further studied.

In our model, cohort simulation was performed until the cohort age reached 79 years or hypothetical death, since life expectancy is 79 years in China. The results of the study showed that screening was more cost-effective in the younger population than in the older population. One-time EC screening targeting individuals aged 50–69 years was not cost-effective. The main reason for this finding was that screening targeting younger individuals resulted in a significant decrease in cumulative EC mortalities. Normal epithelial tissue takes decades to develop into cancer tissue based on its natural progression history. Early detection could result in early treatment, improved quality of life, and saved costs. Precancerous dysplasia is an unstable lesion, and early detection might be helpful for early intervention to reduce the risk of EC development. Screening in younger patients is more likely to detect EC, especially precancerous lesions, in the early natural history progress. Moreover, older patients ae more likely to be diagnosed with other diseases or die of other diseases than younger patients. Treating older patients could increase costs and reduce quality of life due to increases in complications and infections. Moreover, treatment could result in an adverse psychological outcomes and mental stress. All of these findings suggest that it is not necessary to target individuals aged 50–69 years for screening.

In 2016, the screening age was recommended to be extended to 74 years in the urban cancer screening program in China [44]. Although our study did not examine the economic value of EC screening in individuals aged more than 70 years, the results of the study imply that screening targeting elderly people is unlikely to be cost-effective. However, EC screening targeting elderly individuals who are identified by the threshold of the risk function computed from the health risk appraisal questionnaire survey may have economic value. We strongly suggest that economically underdeveloped areas or areas at low risk of EC areas conduct a health risk appraisal survey for EC before clinical screening. Nevertheless, further studies should be performed to test these findings.

One-way sensitivity analyses revealed that the RR and u_LGIN had strong impacts on the cost-effectiveness of EC screening. Increasing the RR from 0.70 to 0.84 could make the screening strategy not cost-effective, indicating that screening is cost-effective only if it significantly reduces the incidence of EC. This result also confirmed the importance and necessity of endoscopic follow-up for LGIN, which could substantially reduce the risk of EC development. Furthermore, assuming that patients with LGIN had the same quality of life as healthy individuals, screening with follow-up targeting individuals aged 45–49 years was not cost-effective, with both higher costs and less QALYs. However, assuming that patients with LGIN had the same health status as healthy individuals was *unlikely* because patients with LGIN could have adverse psychological outcomes and mental stress when a true positive is detected, and these effects could have a negative impact on health.

Some limitations to this study should be considered when illustrating the outcomes. The main limitation was the data sources of EC incidence, detection, cancer stage distributions and costs derived from Zhejiang Province. However, the EC burden in Zhejiang Province could represent the national level very well. Although the current study analyzed Zhejiang-based costs, the sensitivity analyses confirmed the robustness of the model. In addition, our model simulation produced the long-term costs and effectiveness associated with the screening strategies. However, it did not provide accurate evaluations as do randomized clinical controlled trials. Furthermore, the current study may have underestimated the total expected costs because the hidden costs were not computed. However, this would not affect the identification of cost-effectiveness of EC screening, since the ICER was the indicator. Moreover, the sensitivity and specificity of the screening test were not studied since Lugol’s iodine staining plus indicative biopsy is commonly regarded as the gold standard for EC diagnosis [45, 46].

## Conclusion

One-time EC screening targeting individuals aged 40–49 years showed long-term cost-effectiveness in Zhejiang Province. The results of this study indicate the *likely* national cost-effectiveness of EC screening targeting the same age group. More importantly, endoscopic follow-up for LGIN should be performed to strengthen the screening benefit. For individuals over 50 years of age or from economically underdeveloped areas or areas at low risk of EC, the government and policymakers should take comprehensive measures to improve the opportunity for EC screening, including providing a health risk appraisal survey for EC. In addition, EC still poses a huge threat to the worldwide population, and building a solid global EC screening guideline and performing a cost-effectiveness analysis are currently crucial. Hopefully, much work on the topic of global EC screening will be conducted in the future.

## Supplementary information

**Additional file 1.** Additional tables.

## Data Availability

All data generated or analyzed during this study are included in this published article (Additional file 1).

## Abbreviations

EC: Esophageal cancer
ESCC: Esophageal squamous cell carcinoma
LGIN: Low-grade intraepithelial neoplasia
IC: Intraductal carcinoma
SM: Submucosal cancer
Mod: Moderate cancer stage
Adv: Advanced cancer stage
IC_DFS: Disease-free survival state of IC
SM_DFS: Disease-free survival state of SM
Mod_DFS: Disease-free survival state of moderate stage
Adv_PFS: Disease progress-free state of advanced stage
QALYs: Quality-adjusted life-years
ICER: Incremental cost-effectiveness ratio
