# Supplementary material for: Modeling the Cost-effectiveness of Esophageal Cancer Screening in China
Source: Cost Eff Resour Alloc. 2020 Sep 10;18:33. doi: 10.1186/s12962-020-00230-y (PMC7488134; doi:10.1186/s12962-020-00230-y)
Supplement: Supplementary file 1 — Additional file 1. Additional tables. [file 12962_2020_230_MOESM1_ESM.docx]

**Modeling the Cost-effectiveness of Esophageal Cancer Screening in China**

**Supplementary material**

eTable 1-1: Stage cohorts and expected costs in non-screening scenario for 40-44 years

eTable 1-2: Stage cohorts and expected costs in screening without follow-up scenario for 40-44 years

eTable 1-3: Stage cohorts and expected costs in screening with follow-up scenario for 40-44 years

eTable 1-4: Stage cohorts and expected costs in non-screening scenario for 45-49 years

eTable 1-5: Stage cohorts and expected costs in screening without follow-up scenario for 45-49 years

eTable 1-6: Stage cohorts and expected costs in screening with follow-up scenario for 45-49 years

eTable 1-7: Stage cohorts and expected costs in non-screening scenario for 50-54 years

eTable 1-8: Stage cohorts and expected costs in screening without follow-up scenario for 50-54 years

eTable 1-9: Stage cohorts and expected costs in screening with follow-up scenario for 50-54 years

eTable 1-10: Stage cohorts and expected costs in non-screening scenario for 55-59 years

eTable 1-11: Stage cohorts and expected costs in screening without follow-up scenario for 55-59 years

eTable 1-12: Stage cohorts and expected costs in screening with follow-up scenario for 55-59 years

eTable 1-13: Stage cohorts and expected costs in non-screening scenario for 60-64 years

eTable 1-14: Stage cohorts and expected costs in screening without follow-up scenario for 60-64 years

eTable 1-15: Stage cohorts and expected costs in screening with follow-up scenario for 60-64 years

eTable 1-16: Stage cohorts and expected costs in non-screening scenario for 65-69 years

eTable 1-17: Stage cohorts and expected costs in screening without follow-up scenario for 65-69 years

eTable 1-18: Stage cohorts and expected costs in screening with follow-up scenario for 65-69 years

eTable 2: The total expected costs for different Markov states among different screening strategies

eTable 1-1 Stage cohorts and expected costs in non-screening scenario for 40-44 years

| Stage | Normal  /n | LGIN  /n | IC  /n | DFS_  IC/n | SM  /n | DFS_  SM/n | Moderate  /n | DFS_  Mod/n | Advanced  /n | PFS_  Adv/n | Dead  /n | Stage Cost/USD |
| --- | --- | --- | --- | --- | --- | --- | --- | --- | --- | --- | --- | --- |
| 0 | 99999 | 0 | 0 | 0 | 0 | 0 | 1 | 0 | 0 | 0 | 0 | 0.02 |
| 1 | 99875 | 0 | 0 | 0 | 0 | 0 | 1 | 1 | 1 | 0 | 123 | 0.04 |
| 2 | 99751 | 0 | 0 | 0 | 0 | 0 | 1 | 1 | 1 | 0 | 245 | 0.05 |
| 3 | 99628 | 0 | 0 | 0 | 0 | 0 | 1 | 2 | 1 | 0 | 368 | 0.05 |
| 4 | 99504 | 0 | 0 | 0 | 0 | 0 | 1 | 2 | 1 | 0 | 491 | 0.06 |
| 5 | 99381 | 0 | 0 | 0 | 0 | 0 | 1 | 2 | 1 | 0 | 613 | 0.06 |
| 6 | 99193 | 0 | 0 | 0 | 0 | 0 | 4 | 3 | 2 | 0 | 798 | 0.14 |
| 7 | 99005 | 0 | 0 | 0 | 0 | 0 | 4 | 4 | 2 | 0 | 983 | 0.17 |
| 8 | 98817 | 0 | 0 | 0 | 0 | 1 | 5 | 6 | 2 | 1 | 1168 | 0.19 |
| 9 | 98630 | 0 | 0 | 0 | 0 | 1 | 5 | 7 | 3 | 1 | 1353 | 0.20 |
| 10 | 98443 | 0 | 0 | 0 | 0 | 1 | 5 | 8 | 3 | 1 | 1538 | 0.21 |
| 11 | 98095 | 0 | 0 | 0 | 1 | 1 | 8 | 9 | 4 | 1 | 1880 | 0.33 |
| 12 | 97749 | 0 | 0 | 0 | 1 | 1 | 9 | 12 | 5 | 1 | 2221 | 0.37 |
| 13 | 97403 | 0 | 0 | 0 | 1 | 2 | 10 | 14 | 5 | 1 | 2563 | 0.40 |
| 14 | 97059 | 0 | 0 | 0 | 1 | 2 | 10 | 16 | 5 | 1 | 2904 | 0.41 |
| 15 | 96716 | 0 | 0 | 0 | 1 | 2 | 10 | 18 | 6 | 1 | 3244 | 0.42 |
| 16 | 96231 | 0 | 1 | 0 | 1 | 2 | 20 | 20 | 10 | 2 | 3711 | 0.78 |
| 17 | 95750 | 0 | 1 | 0 | 1 | 3 | 23 | 27 | 11 | 2 | 4179 | 0.90 |
| 18 | 95270 | 0 | 1 | 0 | 1 | 4 | 24 | 33 | 13 | 3 | 4647 | 0.96 |
| 19 | 94794 | 0 | 1 | 0 | 2 | 4 | 24 | 39 | 14 | 3 | 5115 | 1.00 |
| 20 | 94319 | 0 | 1 | 1 | 2 | 5 | 25 | 44 | 14 | 4 | 5582 | 1.03 |
| 21 | 93589 | 0 | 1 | 1 | 2 | 5 | 31 | 48 | 17 | 4 | 6296 | 1.27 |
| 22 | 92865 | 0 | 1 | 1 | 2 | 6 | 33 | 54 | 18 | 4 | 7008 | 1.36 |
| 23 | 92146 | 0 | 1 | 1 | 2 | 7 | 34 | 61 | 19 | 5 | 7717 | 1.40 |
| 24 | 91433 | 0 | 1 | 1 | 2 | 7 | 34 | 66 | 20 | 5 | 8423 | 1.43 |
| 25 | 90725 | 0 | 1 | 1 | 2 | 8 | 34 | 70 | 20 | 5 | 9124 | 1.44 |
| 26 | 89538 | 0 | 2 | 1 | 3 | 8 | 41 | 73 | 23 | 6 | 10297 | 1.67 |
| 27 | 88367 | 0 | 2 | 1 | 3 | 9 | 41 | 76 | 22 | 6 | 11462 | 1.68 |
| 28 | 87210 | 0 | 2 | 1 | 3 | 10 | 41 | 79 | 22 | 6 | 12614 | 1.68 |
| 29 | 86069 | 0 | 2 | 1 | 3 | 10 | 41 | 81 | 22 | 6 | 13752 | 1.68 |
| 30 | 84943 | 0 | 2 | 1 | 3 | 10 | 41 | 82 | 22 | 6 | 14876 | 1.67 |
| 31 | 82867 | 0 | 2 | 2 | 3 | 11 | 50 | 83 | 26 | 6 | 16937 | 1.99 |
| 32 | 80841 | 0 | 2 | 2 | 4 | 11 | 52 | 89 | 27 | 7 | 18951 | 2.05 |
| 33 | 78865 | 0 | 2 | 2 | 3 | 12 | 52 | 94 | 27 | 7 | 20919 | 2.06 |
| 34 | 76938 | 0 | 2 | 2 | 3 | 13 | 51 | 98 | 27 | 7 | 22842 | 2.05 |
| 35 | 75057 | 0 | 2 | 2 | 3 | 13 | 50 | 100 | 27 | 7 | 24720 | 2.03 |
| 36 | 71936 | 0 | 2 | 2 | 3 | 13 | 50 | 101 | 27 | 7 | 27841 | 2.00 |
| 37 | 68944 | 0 | 2 | 2 | 3 | 14 | 48 | 101 | 26 | 7 | 30835 | 1.95 |
| 38 | 66076 | 0 | 2 | 2 | 3 | 14 | 47 | 100 | 26 | 7 | 33705 | 1.89 |
| 39 | 63328 | 0 | 2 | 2 | 3 | 14 | 45 | 99 | 25 | 7 | 36458 | 0.91 |
| Total | 3617350 | 0 | 41 | 30 | 67 | 224 | 1007 | 1820 | 546 | 141 | 378501 | 40.02 |

A hypothetical cohort with 100,000 individuals was simulated. n the number of individuals in each Markov states

eTable 1-2 Stage cohorts and expected costs in screening without follow-up scenario for 40-44 years

| Stage | Normal  /n | LGIN  /n | IC  /n | DFS_  IC/n | SM  /n | DFS_  SM/n | Moderate  /n | DFS_  Mod/n | Advanced  /n | PFS_  Adv/n | Dead  /n | Stage Cost/USD |
| --- | --- | --- | --- | --- | --- | --- | --- | --- | --- | --- | --- | --- |
| 0 | 99853 | 131 | 15 | 0 | 0 | 0 | 1 | 0 | 0 | 0 | 0 | 6.18 |
| 1 | 99729 | 130 | 1 | 14 | 0 | 0 | 1 | 1 | 0 | 0 | 123 | 0.07 |
| 2 | 99606 | 130 | 0 | 14 | 0 | 0 | 1 | 1 | 1 | 0 | 245 | 0.06 |
| 3 | 99483 | 130 | 0 | 14 | 0 | 1 | 1 | 1 | 1 | 0 | 368 | 0.07 |
| 4 | 99361 | 130 | 0 | 14 | 0 | 1 | 1 | 2 | 1 | 0 | 491 | 0.07 |
| 5 | 99238 | 130 | 0 | 13 | 0 | 1 | 1 | 2 | 1 | 0 | 613 | 0.07 |
| 6 | 99052 | 129 | 0 | 13 | 0 | 1 | 3 | 2 | 1 | 0 | 798 | 0.13 |
| 7 | 98865 | 129 | 0 | 13 | 0 | 1 | 3 | 3 | 2 | 0 | 982 | 0.15 |
| 8 | 98679 | 129 | 0 | 12 | 0 | 1 | 4 | 5 | 2 | 0 | 1167 | 0.16 |
| 9 | 98494 | 129 | 0 | 12 | 0 | 1 | 4 | 6 | 2 | 0 | 1351 | 0.17 |
| 10 | 98309 | 128 | 0 | 12 | 0 | 2 | 4 | 6 | 2 | 1 | 1536 | 0.17 |
| 11 | 97964 | 128 | 0 | 12 | 1 | 2 | 6 | 7 | 3 | 1 | 1877 | 0.26 |
| 12 | 97621 | 127 | 0 | 12 | 1 | 2 | 7 | 9 | 3 | 1 | 2217 | 0.29 |
| 13 | 97279 | 127 | 0 | 12 | 1 | 2 | 7 | 11 | 4 | 1 | 2557 | 0.31 |
| 14 | 96938 | 126 | 0 | 11 | 1 | 2 | 7 | 12 | 4 | 1 | 2897 | 0.32 |
| 15 | 96598 | 126 | 0 | 11 | 1 | 2 | 7 | 14 | 4 | 1 | 3235 | 0.32 |
| 16 | 96122 | 125 | 1 | 11 | 1 | 3 | 14 | 15 | 7 | 1 | 3701 | 0.57 |
| 17 | 95648 | 125 | 1 | 12 | 1 | 3 | 16 | 19 | 8 | 2 | 4166 | 0.66 |
| 18 | 95176 | 124 | 1 | 12 | 1 | 4 | 17 | 24 | 9 | 2 | 4630 | 0.70 |
| 19 | 94707 | 123 | 1 | 12 | 1 | 4 | 17 | 28 | 10 | 2 | 5094 | 0.73 |
| 20 | 94240 | 123 | 1 | 12 | 1 | 4 | 18 | 32 | 10 | 3 | 5557 | 0.75 |
| 21 | 93521 | 121 | 1 | 12 | 2 | 5 | 22 | 34 | 12 | 3 | 6267 | 0.92 |
| 22 | 92807 | 120 | 1 | 13 | 2 | 5 | 23 | 39 | 13 | 3 | 6973 | 0.97 |
| 23 | 92099 | 119 | 1 | 13 | 2 | 6 | 24 | 43 | 14 | 3 | 7676 | 1.00 |
| 24 | 91396 | 118 | 1 | 14 | 2 | 6 | 24 | 47 | 14 | 4 | 8375 | 1.02 |
| 25 | 90698 | 117 | 1 | 14 | 2 | 6 | 24 | 50 | 14 | 4 | 9069 | 1.03 |
| 26 | 89524 | 116 | 1 | 14 | 2 | 7 | 29 | 52 | 16 | 4 | 10235 | 1.20 |
| 27 | 88365 | 114 | 1 | 15 | 2 | 7 | 29 | 54 | 16 | 4 | 11392 | 1.20 |
| 28 | 87221 | 113 | 1 | 15 | 2 | 7 | 29 | 56 | 16 | 4 | 12535 | 1.20 |
| 29 | 86092 | 111 | 1 | 16 | 2 | 8 | 29 | 57 | 16 | 4 | 13664 | 1.19 |
| 30 | 84977 | 110 | 1 | 16 | 2 | 8 | 29 | 58 | 16 | 4 | 14779 | 1.19 |
| 31 | 82916 | 107 | 2 | 16 | 3 | 8 | 36 | 59 | 18 | 4 | 16831 | 1.41 |
| 32 | 80906 | 104 | 2 | 17 | 3 | 9 | 37 | 63 | 19 | 5 | 18837 | 1.46 |
| 33 | 78944 | 101 | 2 | 17 | 3 | 9 | 37 | 67 | 19 | 5 | 20797 | 1.46 |
| 34 | 77029 | 99 | 2 | 18 | 3 | 9 | 36 | 69 | 19 | 5 | 22711 | 1.46 |
| 35 | 75161 | 96 | 1 | 18 | 3 | 10 | 36 | 71 | 19 | 5 | 24580 | 1.44 |
| 36 | 72050 | 92 | 1 | 18 | 3 | 10 | 35 | 71 | 19 | 5 | 27696 | 1.42 |
| 37 | 69067 | 88 | 1 | 18 | 2 | 10 | 34 | 71 | 19 | 5 | 30684 | 1.38 |
| 38 | 66208 | 85 | 1 | 18 | 2 | 10 | 33 | 71 | 18 | 5 | 33549 | 1.34 |
| 39 | 63467 | 81 | 1 | 18 | 2 | 10 | 32 | 70 | 17 | 5 | 36297 | 0.65 |
| Total | 3615410 | 4694 | 47 | 547 | 54 | 188 | 719 | 1303 | 387 | 100 | 376553 | 35.14 |

A hypothetical cohort with 100,000 individuals was simulated. n the number of individuals in each Markov states

eTable 1-3 Stage cohorts and expected costs in screening with follow-up scenario for 40-44 years

| Stage | Normal  /n | LGIN  /n | IC  /n | DFS_  IC/n | SM  /n | DFS_  SM/n | Moderate  /n | DFS_  Mod/n | Advanced  /n | PFS_  Adv/n | Dead  /n | Stage Cost/USD |
| --- | --- | --- | --- | --- | --- | --- | --- | --- | --- | --- | --- | --- |
| 0 | 99853 | 131 | 15 | 0 | 0 | 0 | 1 | 0 | 0 | 0 | 0 | 6.20 |
| 1 | 99748 | 112 | 1 | 1 | 0 | 0 | 1 | 1 | 0 | 0 | 123 | 0.08 |
| 2 | 99641 | 96 | 0 | 1 | 0 | 0 | 1 | 1 | 0 | 0 | 245 | 0.08 |
| 3 | 99532 | 82 | 0 | 1 | 0 | 1 | 1 | 1 | 1 | 0 | 368 | 0.08 |
| 4 | 99421 | 70 | 0 | 1 | 0 | 1 | 1 | 2 | 1 | 0 | 491 | 0.08 |
| 5 | 99308 | 60 | 0 | 1 | 0 | 1 | 1 | 2 | 1 | 0 | 613 | 0.08 |
| 6 | 99130 | 51 | 0 | 1 | 0 | 1 | 3 | 2 | 1 | 0 | 798 | 0.14 |
| 7 | 98951 | 44 | 0 | 1 | 0 | 1 | 3 | 3 | 2 | 0 | 982 | 0.16 |
| 8 | 98771 | 38 | 0 | 1 | 0 | 1 | 4 | 5 | 2 | 0 | 1167 | 0.17 |
| 9 | 98590 | 32 | 0 | 1 | 0 | 1 | 4 | 6 | 2 | 0 | 1351 | 0.17 |
| 10 | 98410 | 27 | 0 | 1 | 0 | 2 | 4 | 6 | 2 | 1 | 1536 | 0.18 |
| 11 | 98069 | 23 | 0 | 1 | 1 | 2 | 6 | 7 | 3 | 1 | 1877 | 0.26 |
| 12 | 97728 | 20 | 0 | 1 | 1 | 2 | 7 | 9 | 3 | 1 | 2217 | 0.29 |
| 13 | 97389 | 17 | 0 | 1 | 1 | 2 | 7 | 11 | 4 | 1 | 2557 | 0.31 |
| 14 | 97050 | 15 | 0 | 1 | 1 | 2 | 7 | 12 | 4 | 1 | 2897 | 0.32 |
| 15 | 96712 | 12 | 0 | 1 | 1 | 2 | 7 | 13 | 4 | 1 | 3235 | 0.32 |
| 16 | 96237 | 11 | 1 | 1 | 1 | 3 | 14 | 14 | 7 | 1 | 3700 | 0.57 |
| 17 | 95764 | 9 | 1 | 1 | 1 | 3 | 16 | 19 | 8 | 2 | 4165 | 0.66 |
| 18 | 95293 | 8 | 1 | 1 | 1 | 4 | 17 | 24 | 9 | 2 | 4630 | 0.70 |
| 19 | 94824 | 7 | 1 | 1 | 1 | 4 | 17 | 28 | 10 | 2 | 5094 | 0.73 |
| 20 | 94358 | 6 | 1 | 1 | 1 | 4 | 17 | 32 | 10 | 3 | 5556 | 0.75 |
| 21 | 93638 | 5 | 1 | 1 | 2 | 5 | 22 | 34 | 12 | 3 | 6266 | 0.91 |
| 22 | 92924 | 4 | 1 | 1 | 2 | 5 | 23 | 39 | 13 | 3 | 6973 | 0.97 |
| 23 | 92216 | 3 | 1 | 1 | 2 | 6 | 24 | 43 | 13 | 3 | 7675 | 1.00 |
| 24 | 91512 | 3 | 1 | 1 | 2 | 6 | 24 | 47 | 14 | 4 | 8374 | 1.02 |
| 25 | 90814 | 2 | 1 | 1 | 2 | 6 | 24 | 49 | 14 | 4 | 9069 | 1.03 |
| 26 | 89639 | 2 | 1 | 1 | 2 | 7 | 29 | 52 | 16 | 4 | 10235 | 1.19 |
| 27 | 88479 | 2 | 1 | 1 | 2 | 7 | 29 | 54 | 16 | 4 | 11391 | 1.20 |
| 28 | 87333 | 1 | 1 | 2 | 2 | 7 | 29 | 56 | 16 | 4 | 12534 | 1.20 |
| 29 | 86203 | 1 | 1 | 2 | 2 | 8 | 29 | 57 | 16 | 4 | 13663 | 1.19 |
| 30 | 85087 | 1 | 1 | 2 | 2 | 8 | 29 | 58 | 16 | 4 | 14778 | 1.18 |
| 31 | 83024 | 1 | 2 | 2 | 3 | 8 | 36 | 59 | 18 | 4 | 16830 | 1.41 |
| 32 | 81011 | 1 | 2 | 2 | 3 | 9 | 37 | 63 | 19 | 5 | 18836 | 1.45 |
| 33 | 79046 | 1 | 2 | 2 | 3 | 9 | 37 | 66 | 19 | 5 | 20796 | 1.46 |
| 34 | 77129 | 1 | 2 | 2 | 3 | 9 | 36 | 69 | 19 | 5 | 22710 | 1.45 |
| 35 | 75259 | 0 | 1 | 2 | 3 | 10 | 36 | 70 | 19 | 5 | 24578 | 1.43 |
| 36 | 72144 | 0 | 1 | 2 | 2 | 10 | 35 | 71 | 19 | 5 | 27694 | 1.42 |
| 37 | 69157 | 0 | 1 | 2 | 2 | 10 | 34 | 71 | 19 | 5 | 30682 | 1.38 |
| 38 | 66294 | 0 | 1 | 2 | 2 | 10 | 33 | 70 | 18 | 5 | 33548 | 1.33 |
| 39 | 63550 | 0 | 1 | 2 | 2 | 10 | 32 | 69 | 17 | 5 | 36295 | 0.64 |
| Total | 3619235 | 899 | 47 | 55 | 54 | 187 | 716 | 1297 | 385 | 99 | 376531 | 35.17 |

A hypothetical cohort with 100,000 individuals was simulated. n the number of individuals in each Markov states

eTable 1-4 Stage cohorts and expected costs in non-screening scenario for 45-49 years

| Stage | Normal  /n | LGIN  /n | IC  /n | DFS_  IC/n | SM  /n | DFS_  SM/n | Moderate  /n | DFS_  Mod/n | Advanced  /n | PFS_  Adv/n | Dead  /n | Stage Cost/USD |
| --- | --- | --- | --- | --- | --- | --- | --- | --- | --- | --- | --- | --- |
| 0 | 99995 | 0 | 0 | 0 | 0 | 0 | 3 | 0 | 1 | 0 | 0 | 0.06 |
| 1 | 99806 | 0 | 0 | 0 | 0 | 0 | 4 | 2 | 2 | 0 | 185 | 0.16 |
| 2 | 99616 | 0 | 0 | 0 | 0 | 0 | 4 | 4 | 2 | 0 | 371 | 0.18 |
| 3 | 99428 | 0 | 0 | 1 | 0 | 1 | 5 | 6 | 2 | 1 | 557 | 0.19 |
| 4 | 99239 | 0 | 0 | 1 | 0 | 1 | 5 | 7 | 3 | 1 | 744 | 0.20 |
| 5 | 99051 | 0 | 0 | 1 | 0 | 1 | 5 | 8 | 3 | 1 | 930 | 0.21 |
| 6 | 98701 | 0 | 0 | 1 | 1 | 1 | 8 | 9 | 4 | 1 | 1274 | 0.34 |
| 7 | 98352 | 0 | 0 | 1 | 1 | 1 | 9 | 12 | 5 | 1 | 1618 | 0.38 |
| 8 | 98005 | 0 | 0 | 2 | 1 | 2 | 10 | 14 | 5 | 1 | 1961 | 0.40 |
| 9 | 97658 | 0 | 0 | 2 | 1 | 2 | 10 | 16 | 6 | 1 | 2304 | 0.42 |
| 10 | 97313 | 0 | 0 | 2 | 1 | 2 | 10 | 18 | 6 | 2 | 2646 | 0.43 |
| 11 | 96826 | 0 | 1 | 2 | 1 | 2 | 20 | 20 | 10 | 2 | 3116 | 0.79 |
| 12 | 96341 | 0 | 1 | 3 | 1 | 3 | 23 | 27 | 12 | 2 | 3587 | 0.92 |
| 13 | 95859 | 0 | 1 | 4 | 1 | 4 | 24 | 34 | 13 | 3 | 4058 | 0.98 |
| 14 | 95379 | 0 | 1 | 5 | 2 | 4 | 24 | 40 | 14 | 3 | 4529 | 1.03 |
| 15 | 94902 | 0 | 1 | 6 | 2 | 5 | 25 | 44 | 14 | 4 | 4999 | 1.05 |
| 16 | 94167 | 0 | 1 | 6 | 2 | 5 | 31 | 48 | 17 | 4 | 5718 | 1.30 |
| 17 | 93438 | 0 | 1 | 7 | 2 | 6 | 33 | 55 | 18 | 4 | 6434 | 1.38 |
| 18 | 92715 | 0 | 1 | 8 | 2 | 7 | 34 | 61 | 19 | 5 | 7147 | 1.43 |
| 19 | 91998 | 0 | 1 | 9 | 2 | 7 | 34 | 66 | 20 | 5 | 7857 | 1.46 |
| 20 | 91286 | 0 | 1 | 10 | 2 | 8 | 34 | 70 | 20 | 5 | 8563 | 1.47 |
| 21 | 90091 | 0 | 2 | 11 | 3 | 8 | 41 | 73 | 23 | 6 | 9743 | 1.71 |
| 22 | 88912 | 0 | 2 | 12 | 3 | 9 | 41 | 77 | 22 | 6 | 10916 | 1.72 |
| 23 | 87749 | 0 | 2 | 13 | 3 | 10 | 41 | 79 | 22 | 6 | 12075 | 1.72 |
| 24 | 86601 | 0 | 2 | 14 | 3 | 10 | 41 | 81 | 22 | 6 | 13220 | 1.71 |
| 25 | 85468 | 0 | 2 | 15 | 3 | 10 | 41 | 83 | 22 | 6 | 14351 | 1.70 |
| 26 | 83379 | 0 | 2 | 15 | 4 | 11 | 51 | 83 | 26 | 6 | 16424 | 2.03 |
| 27 | 81341 | 0 | 2 | 16 | 4 | 12 | 52 | 89 | 27 | 7 | 18450 | 2.10 |
| 28 | 79352 | 0 | 2 | 17 | 4 | 12 | 52 | 95 | 27 | 7 | 20431 | 2.11 |
| 29 | 77413 | 0 | 2 | 18 | 3 | 13 | 52 | 98 | 28 | 7 | 22366 | 2.09 |
| 30 | 75521 | 0 | 2 | 19 | 3 | 13 | 51 | 100 | 27 | 8 | 24255 | 2.07 |
| 31 | 72380 | 0 | 2 | 20 | 3 | 13 | 50 | 101 | 27 | 7 | 27396 | 2.04 |
| 32 | 69369 | 0 | 2 | 20 | 3 | 14 | 49 | 101 | 26 | 7 | 30408 | 1.99 |
| 33 | 66484 | 0 | 2 | 20 | 3 | 14 | 47 | 101 | 26 | 7 | 33296 | 1.93 |
| 34 | 63719 | 0 | 2 | 20 | 3 | 14 | 45 | 99 | 25 | 7 | 36065 | 0.93 |
| Total | 3137853 | 0 | 41 | 302 | 67 | 224 | 1010 | 1822 | 547 | 141 | 357992 | 40.63 |

A hypothetical cohort with 100,000 individuals was simulated. n the number of individuals in each Markov states

eTable 1-5 Stage cohorts and expected costs in screening without follow-up scenario for 45-49 years

| Stage | Normal  /n | LGIN  /n | IC  /n | DFS_  IC/n | SM  /n | DFS_  SM/n | Moderate  /n | DFS_  Mod/n | Advanced  /n | PFS_  Adv/n | Dead  /n | Stage Cost/USD |
| --- | --- | --- | --- | --- | --- | --- | --- | --- | --- | --- | --- | --- |
| 0 | 99384 | 545 | 62 | 0 | 2 | 0 | 5 | 0 | 2 | 0 | 0 | 6.67 |
| 1 | 99197 | 544 | 4 | 58 | 0 | 2 | 4 | 3 | 2 | 0 | 186 | 0.27 |
| 2 | 99011 | 543 | 1 | 60 | 1 | 2 | 4 | 4 | 2 | 0 | 372 | 0.25 |
| 3 | 98825 | 542 | 1 | 58 | 1 | 2 | 4 | 6 | 2 | 1 | 558 | 0.26 |
| 4 | 98639 | 541 | 1 | 57 | 1 | 3 | 5 | 7 | 2 | 1 | 744 | 0.26 |
| 5 | 98453 | 540 | 1 | 55 | 1 | 4 | 5 | 8 | 2 | 1 | 931 | 0.27 |
| 6 | 98108 | 537 | 1 | 54 | 1 | 4 | 7 | 9 | 3 | 1 | 1274 | 0.36 |
| 7 | 97765 | 535 | 1 | 52 | 1 | 4 | 8 | 11 | 4 | 1 | 1618 | 0.39 |
| 8 | 97422 | 533 | 1 | 51 | 1 | 5 | 8 | 13 | 4 | 1 | 1961 | 0.41 |
| 9 | 97080 | 531 | 1 | 50 | 1 | 5 | 8 | 15 | 4 | 1 | 2303 | 0.42 |
| 10 | 96740 | 529 | 1 | 49 | 1 | 5 | 8 | 16 | 4 | 1 | 2644 | 0.42 |
| 11 | 96263 | 526 | 1 | 47 | 2 | 6 | 16 | 17 | 7 | 1 | 3113 | 0.68 |
| 12 | 95789 | 523 | 1 | 46 | 2 | 6 | 18 | 22 | 9 | 2 | 3582 | 0.77 |
| 13 | 95316 | 521 | 1 | 46 | 2 | 7 | 18 | 27 | 9 | 2 | 4050 | 0.81 |
| 14 | 94847 | 518 | 1 | 45 | 2 | 7 | 19 | 31 | 10 | 3 | 4518 | 0.84 |
| 15 | 94379 | 515 | 1 | 44 | 2 | 8 | 19 | 35 | 10 | 3 | 4985 | 0.86 |
| 16 | 93659 | 510 | 1 | 43 | 2 | 8 | 24 | 37 | 12 | 3 | 5700 | 1.03 |
| 17 | 92944 | 506 | 1 | 43 | 2 | 8 | 25 | 42 | 13 | 3 | 6412 | 1.08 |
| 18 | 92234 | 502 | 1 | 42 | 2 | 9 | 25 | 47 | 14 | 4 | 7120 | 1.11 |
| 19 | 91530 | 498 | 1 | 41 | 2 | 9 | 25 | 50 | 14 | 4 | 7824 | 1.13 |
| 20 | 90832 | 493 | 1 | 41 | 2 | 10 | 26 | 53 | 15 | 4 | 8524 | 1.14 |
| 21 | 89656 | 487 | 1 | 40 | 3 | 10 | 30 | 55 | 17 | 4 | 9698 | 1.30 |
| 22 | 88495 | 480 | 1 | 40 | 3 | 10 | 31 | 57 | 16 | 4 | 10863 | 1.30 |
| 23 | 87349 | 473 | 1 | 39 | 3 | 10 | 30 | 59 | 16 | 4 | 12014 | 1.30 |
| 24 | 86219 | 467 | 1 | 39 | 3 | 10 | 30 | 60 | 16 | 4 | 13150 | 1.29 |
| 25 | 85102 | 460 | 1 | 38 | 3 | 11 | 30 | 61 | 16 | 4 | 14273 | 1.28 |
| 26 | 83039 | 449 | 2 | 37 | 3 | 11 | 37 | 61 | 19 | 4 | 16339 | 1.51 |
| 27 | 81025 | 437 | 2 | 37 | 3 | 11 | 38 | 66 | 19 | 5 | 18358 | 1.55 |
| 28 | 79060 | 426 | 2 | 36 | 3 | 11 | 38 | 69 | 20 | 5 | 20330 | 1.56 |
| 29 | 77143 | 415 | 2 | 36 | 3 | 12 | 37 | 72 | 20 | 5 | 22256 | 1.55 |
| 30 | 75272 | 405 | 2 | 35 | 3 | 12 | 37 | 73 | 20 | 5 | 24137 | 1.53 |
| 31 | 72156 | 387 | 2 | 34 | 3 | 12 | 36 | 74 | 19 | 5 | 27271 | 1.51 |
| 32 | 69169 | 371 | 2 | 33 | 3 | 12 | 35 | 74 | 19 | 5 | 30278 | 1.46 |
| 33 | 66305 | 355 | 1 | 32 | 3 | 12 | 34 | 73 | 18 | 5 | 33161 | 1.42 |
| 34 | 63561 | 340 | 1 | 31 | 3 | 12 | 33 | 72 | 18 | 5 | 35925 | 0.68 |
| Total | 3121970 | 16983 | 105 | 1488 | 74 | 270 | 758 | 1381 | 399 | 103 | 356470 | 38.65 |

A hypothetical cohort with 100,000 individuals was simulated. n the number of individuals in each Markov states

eTable 1-6 Stage cohorts and expected costs in screening with follow-up scenario for 45-49 years

| Stage | Normal  /n | LGIN  /n | IC  /n | DFS_  IC/n | SM  /n | DFS_  SM/n | Moderate  /n | DFS_  Mod/n | Advanced  /n | PFS_  Adv/n | Dead  /n | Stage Cost/USD |
| --- | --- | --- | --- | --- | --- | --- | --- | --- | --- | --- | --- | --- |
| 0 | 99384 | 545 | 62 | 0 | 2 | 0 | 5 | 0 | 2 | 0 | 0 | 6.76 |
| 1 | 99275 | 466 | 4 | 58 | 0 | 2 | 4 | 3 | 2 | 0 | 186 | 0.34 |
| 2 | 99155 | 399 | 1 | 60 | 1 | 2 | 4 | 4 | 2 | 0 | 372 | 0.31 |
| 3 | 99026 | 341 | 1 | 59 | 1 | 2 | 4 | 6 | 2 | 1 | 558 | 0.31 |
| 4 | 98888 | 292 | 1 | 57 | 1 | 3 | 5 | 7 | 2 | 1 | 744 | 0.31 |
| 5 | 98744 | 249 | 1 | 56 | 1 | 4 | 5 | 8 | 2 | 1 | 931 | 0.30 |
| 6 | 98433 | 213 | 1 | 54 | 1 | 4 | 7 | 9 | 3 | 1 | 1274 | 0.39 |
| 7 | 98119 | 182 | 1 | 53 | 1 | 4 | 8 | 11 | 4 | 1 | 1617 | 0.41 |
| 8 | 97801 | 155 | 1 | 52 | 1 | 5 | 8 | 13 | 4 | 1 | 1960 | 0.42 |
| 9 | 97480 | 132 | 1 | 50 | 1 | 5 | 8 | 14 | 4 | 1 | 2302 | 0.43 |
| 10 | 97157 | 113 | 1 | 49 | 1 | 5 | 8 | 16 | 4 | 1 | 2644 | 0.43 |
| 11 | 96694 | 96 | 1 | 48 | 2 | 6 | 15 | 17 | 7 | 1 | 3113 | 0.68 |
| 12 | 96231 | 82 | 1 | 47 | 2 | 6 | 17 | 22 | 8 | 2 | 3581 | 0.77 |
| 13 | 95769 | 70 | 1 | 46 | 2 | 7 | 18 | 27 | 9 | 2 | 4049 | 0.81 |
| 14 | 95307 | 59 | 1 | 45 | 2 | 7 | 18 | 31 | 10 | 2 | 4517 | 0.83 |
| 15 | 94845 | 51 | 1 | 45 | 2 | 8 | 19 | 34 | 10 | 3 | 4983 | 0.85 |
| 16 | 94128 | 43 | 1 | 44 | 2 | 8 | 23 | 37 | 12 | 3 | 5698 | 1.02 |
| 17 | 93416 | 36 | 1 | 43 | 2 | 8 | 24 | 41 | 13 | 3 | 6410 | 1.07 |
| 18 | 92708 | 31 | 1 | 43 | 2 | 9 | 25 | 46 | 14 | 4 | 7118 | 1.10 |
| 19 | 92005 | 26 | 1 | 42 | 2 | 9 | 25 | 49 | 14 | 4 | 7821 | 1.12 |
| 20 | 91307 | 22 | 1 | 41 | 2 | 10 | 25 | 52 | 14 | 4 | 8521 | 1.13 |
| 21 | 90128 | 19 | 1 | 41 | 3 | 10 | 30 | 54 | 16 | 4 | 9695 | 1.29 |
| 22 | 88964 | 16 | 1 | 40 | 3 | 10 | 30 | 56 | 16 | 4 | 10859 | 1.29 |
| 23 | 87814 | 13 | 1 | 40 | 3 | 10 | 30 | 58 | 16 | 4 | 12010 | 1.28 |
| 24 | 86679 | 11 | 1 | 39 | 3 | 10 | 30 | 60 | 16 | 4 | 13146 | 1.28 |
| 25 | 85559 | 10 | 1 | 39 | 3 | 11 | 30 | 60 | 16 | 4 | 14269 | 1.27 |
| 26 | 83485 | 8 | 2 | 38 | 3 | 11 | 36 | 61 | 18 | 4 | 16334 | 1.49 |
| 27 | 81462 | 7 | 2 | 37 | 3 | 11 | 37 | 65 | 19 | 5 | 18353 | 1.54 |
| 28 | 79487 | 5 | 2 | 37 | 3 | 11 | 37 | 68 | 19 | 5 | 20324 | 1.54 |
| 29 | 77560 | 5 | 2 | 36 | 3 | 12 | 37 | 71 | 19 | 5 | 22250 | 1.53 |
| 30 | 75680 | 4 | 2 | 35 | 3 | 12 | 36 | 72 | 19 | 5 | 24131 | 1.51 |
| 31 | 72548 | 3 | 2 | 34 | 3 | 12 | 36 | 73 | 19 | 5 | 27266 | 1.49 |
| 32 | 69545 | 3 | 2 | 33 | 3 | 12 | 35 | 73 | 19 | 5 | 30272 | 1.45 |
| 33 | 66666 | 2 | 1 | 32 | 3 | 12 | 34 | 72 | 18 | 5 | 33155 | 1.40 |
| 34 | 63907 | 2 | 1 | 31 | 3 | 12 | 32 | 71 | 18 | 5 | 35919 | 0.67 |
| Total | 3135356 | 3711 | 106 | 1502 | 74 | 268 | 746 | 1360 | 393 | 101 | 356383 | 38.78 |

A hypothetical cohort with 100,000 individuals was simulated. n the number of individuals in each Markov states

eTable 1-7 Stage cohorts and expected costs in non-screening scenario for 50-54 years

| Stage | Normal  /n | LGIN  /n | IC  /n | DFS_  IC/n | SM  /n | DFS_  SM/n | Moderate  /n | DFS_  Mod/n | Advanced  /n | PFS_  Adv/n | Dead  /n | Stage Cost/USD |
| --- | --- | --- | --- | --- | --- | --- | --- | --- | --- | --- | --- | --- |
| 0 | 99990 | 0 | 0 | 0 | 0 | 0 | 7 | 0 | 3 | 0 | 0 | 0.12 |
| 1 | 99637 | 0 | 0 | 0 | 1 | 0 | 8 | 4 | 4 | 0 | 345 | 0.32 |
| 2 | 99284 | 0 | 0 | 1 | 1 | 1 | 9 | 8 | 4 | 1 | 691 | 0.37 |
| 3 | 98933 | 0 | 0 | 1 | 1 | 1 | 9 | 11 | 5 | 1 | 1037 | 0.39 |
| 4 | 98584 | 0 | 0 | 1 | 1 | 1 | 10 | 14 | 5 | 1 | 1382 | 0.41 |
| 5 | 98235 | 0 | 0 | 2 | 1 | 2 | 10 | 16 | 6 | 1 | 1727 | 0.43 |
| 6 | 97744 | 0 | 1 | 2 | 1 | 2 | 20 | 18 | 10 | 2 | 2201 | 0.80 |
| 7 | 97254 | 0 | 1 | 3 | 1 | 3 | 23 | 26 | 12 | 2 | 2675 | 0.93 |
| 8 | 96767 | 0 | 1 | 4 | 1 | 4 | 24 | 33 | 13 | 3 | 3151 | 1.00 |
| 9 | 96283 | 0 | 1 | 4 | 2 | 4 | 24 | 39 | 14 | 3 | 3626 | 1.04 |
| 10 | 95801 | 0 | 1 | 5 | 2 | 5 | 25 | 44 | 14 | 4 | 4100 | 1.07 |
| 11 | 95060 | 0 | 1 | 6 | 2 | 5 | 31 | 48 | 17 | 4 | 4826 | 1.32 |
| 12 | 94324 | 0 | 1 | 7 | 2 | 6 | 33 | 55 | 19 | 5 | 5549 | 1.41 |
| 13 | 93594 | 0 | 1 | 8 | 2 | 7 | 34 | 61 | 19 | 5 | 6268 | 1.46 |
| 14 | 92870 | 0 | 1 | 9 | 2 | 7 | 34 | 66 | 20 | 5 | 6985 | 1.49 |
| 15 | 92151 | 0 | 1 | 10 | 2 | 8 | 35 | 70 | 20 | 5 | 7697 | 1.51 |
| 16 | 90945 | 0 | 2 | 10 | 3 | 8 | 41 | 74 | 23 | 6 | 8888 | 1.75 |
| 17 | 89755 | 0 | 2 | 11 | 3 | 9 | 42 | 77 | 23 | 6 | 10072 | 1.76 |
| 18 | 88581 | 0 | 2 | 13 | 3 | 10 | 42 | 80 | 23 | 6 | 11242 | 1.76 |
| 19 | 87422 | 0 | 2 | 14 | 3 | 10 | 42 | 82 | 23 | 6 | 12398 | 1.75 |
| 20 | 86278 | 0 | 2 | 14 | 3 | 10 | 41 | 83 | 22 | 6 | 13539 | 1.74 |
| 21 | 84169 | 0 | 2 | 15 | 4 | 11 | 51 | 84 | 26 | 6 | 15632 | 2.07 |
| 22 | 82112 | 0 | 2 | 16 | 4 | 12 | 53 | 90 | 27 | 7 | 17678 | 2.14 |
| 23 | 80105 | 0 | 2 | 17 | 4 | 12 | 53 | 95 | 28 | 7 | 19677 | 2.15 |
| 24 | 78147 | 0 | 2 | 18 | 4 | 13 | 52 | 99 | 28 | 8 | 21630 | 2.14 |
| 25 | 76237 | 0 | 2 | 19 | 3 | 13 | 51 | 101 | 28 | 8 | 23538 | 2.12 |
| 26 | 73066 | 0 | 2 | 20 | 3 | 13 | 51 | 102 | 27 | 7 | 26708 | 2.09 |
| 27 | 70027 | 0 | 2 | 20 | 3 | 14 | 49 | 102 | 27 | 7 | 29748 | 2.04 |
| 28 | 67114 | 0 | 2 | 20 | 3 | 14 | 48 | 102 | 26 | 7 | 32664 | 1.97 |
| 29 | 64323 | 0 | 2 | 20 | 3 | 14 | 46 | 100 | 25 | 7 | 35459 | 0.95 |
| Total | 2664790 | 0 | 41 | 291 | 66 | 219 | 997 | 1786 | 539 | 139 | 331132 | 40.52 |

A hypothetical cohort with 100,000 individuals was simulated. n the number of individuals in each Markov states

eTable 1-8 Stage cohorts and expected costs in screening without scenario for 50-54 years

| Stage | Normal  /n | LGIN  /n | IC  /n | DFS_  IC/n | SM  /n | DFS_  SM/n | Moderate  /n | DFS_  Mod/n | Advanced  /n | PFS_  Adv/n | Dead  /n | Stage Cost/USD |
| --- | --- | --- | --- | --- | --- | --- | --- | --- | --- | --- | --- | --- |
| 0 | 98314 | 1492 | 171 | 0 | 5 | 0 | 13 | 0 | 5 | 0 | 0 | 7.80 |
| 1 | 97970 | 1487 | 11 | 159 | 1 | 4 | 9 | 8 | 4 | 1 | 347 | 0.68 |
| 2 | 97626 | 1481 | 2 | 163 | 3 | 4 | 10 | 11 | 4 | 1 | 694 | 0.61 |
| 3 | 97284 | 1475 | 2 | 159 | 3 | 6 | 10 | 14 | 5 | 1 | 1041 | 0.62 |
| 4 | 96943 | 1470 | 1 | 154 | 3 | 8 | 11 | 17 | 5 | 1 | 1387 | 0.63 |
| 5 | 96603 | 1464 | 1 | 150 | 3 | 9 | 11 | 19 | 5 | 1 | 1733 | 0.64 |
| 6 | 96127 | 1456 | 2 | 146 | 4 | 10 | 18 | 21 | 8 | 1 | 2207 | 0.92 |
| 7 | 95653 | 1448 | 2 | 142 | 4 | 12 | 20 | 26 | 9 | 2 | 2682 | 1.01 |
| 8 | 95182 | 1440 | 2 | 138 | 4 | 13 | 21 | 32 | 10 | 2 | 3156 | 1.05 |
| 9 | 94712 | 1432 | 2 | 134 | 4 | 14 | 22 | 37 | 11 | 3 | 3630 | 1.08 |
| 10 | 94246 | 1424 | 2 | 131 | 4 | 15 | 22 | 41 | 11 | 3 | 4103 | 1.10 |
| 11 | 93526 | 1412 | 2 | 127 | 4 | 15 | 27 | 44 | 13 | 3 | 4827 | 1.28 |
| 12 | 92812 | 1400 | 2 | 123 | 4 | 16 | 28 | 49 | 14 | 4 | 5548 | 1.33 |
| 13 | 92104 | 1388 | 2 | 120 | 4 | 16 | 29 | 54 | 15 | 4 | 6265 | 1.36 |
| 14 | 91401 | 1376 | 2 | 117 | 4 | 17 | 29 | 57 | 15 | 4 | 6978 | 1.38 |
| 15 | 90703 | 1365 | 2 | 114 | 4 | 17 | 29 | 60 | 16 | 4 | 7686 | 1.38 |
| 16 | 89529 | 1346 | 2 | 110 | 4 | 17 | 33 | 63 | 18 | 4 | 8873 | 1.54 |
| 17 | 88370 | 1327 | 2 | 107 | 4 | 17 | 34 | 65 | 17 | 5 | 10052 | 1.54 |
| 18 | 87226 | 1309 | 2 | 104 | 4 | 17 | 34 | 66 | 17 | 5 | 11216 | 1.52 |
| 19 | 86097 | 1291 | 2 | 101 | 4 | 17 | 33 | 67 | 17 | 5 | 12366 | 1.51 |
| 20 | 84982 | 1273 | 2 | 98 | 4 | 17 | 33 | 68 | 17 | 5 | 13501 | 1.49 |
| 21 | 82921 | 1241 | 2 | 94 | 4 | 17 | 40 | 68 | 20 | 5 | 15588 | 1.72 |
| 22 | 80910 | 1209 | 2 | 91 | 4 | 17 | 41 | 72 | 20 | 5 | 17627 | 1.76 |
| 23 | 78948 | 1178 | 2 | 88 | 4 | 18 | 41 | 75 | 21 | 5 | 19620 | 1.76 |
| 24 | 77034 | 1148 | 2 | 84 | 4 | 18 | 40 | 78 | 21 | 6 | 21565 | 1.74 |
| 25 | 75166 | 1119 | 2 | 82 | 4 | 18 | 40 | 79 | 21 | 6 | 23465 | 1.72 |
| 26 | 72054 | 1071 | 2 | 77 | 4 | 17 | 39 | 80 | 20 | 6 | 26629 | 1.69 |
| 27 | 69071 | 1026 | 2 | 73 | 4 | 17 | 38 | 79 | 20 | 6 | 29664 | 1.64 |
| 28 | 66212 | 982 | 2 | 70 | 4 | 17 | 37 | 79 | 19 | 5 | 32574 | 1.58 |
| 29 | 63471 | 940 | 2 | 66 | 4 | 16 | 35 | 77 | 19 | 5 | 35365 | 0.76 |
| Total | 2623198 | 39470 | 234 | 3322 | 113 | 419 | 824 | 1506 | 418 | 108 | 330389 | 44.85 |

A hypothetical cohort with 100,000 individuals was simulated. n the number of individuals in each Markov states

eTable 1-9 Stage cohorts and expected costs in screening with follow-up scenario for 50-54 years

| Stage | Normal  /n | LGIN  /n | IC  /n | DFS_  IC/n | SM  /n | DFS_  SM/n | Moderate  /n | DFS_  Mod/n | Advanced  /n | PFS_  Adv/n | Dead  /n | Stage Cost/USD |
| --- | --- | --- | --- | --- | --- | --- | --- | --- | --- | --- | --- | --- |
| 0 | 98314 | 1492 | 171 | 0 | 5 | 0 | 13 | 0 | 5 | 0 | 0 | 8.02 |
| 1 | 98183 | 1274 | 11 | 159 | 1 | 4 | 8 | 8 | 4 | 1 | 347 | 0.87 |
| 2 | 98020 | 1087 | 2 | 164 | 3 | 4 | 9 | 11 | 4 | 1 | 694 | 0.76 |
| 3 | 97832 | 928 | 2 | 160 | 3 | 6 | 10 | 14 | 4 | 1 | 1040 | 0.75 |
| 4 | 97621 | 792 | 2 | 156 | 3 | 8 | 10 | 16 | 4 | 1 | 1386 | 0.74 |
| 5 | 97392 | 676 | 2 | 151 | 3 | 9 | 10 | 18 | 5 | 1 | 1732 | 0.73 |
| 6 | 97008 | 576 | 2 | 147 | 4 | 10 | 18 | 20 | 7 | 1 | 2206 | 0.98 |
| 7 | 96612 | 490 | 2 | 144 | 4 | 12 | 20 | 25 | 9 | 2 | 2680 | 1.05 |
| 8 | 96206 | 418 | 2 | 140 | 4 | 13 | 20 | 31 | 10 | 2 | 3154 | 1.09 |
| 9 | 95791 | 356 | 2 | 137 | 4 | 14 | 21 | 35 | 10 | 3 | 3628 | 1.10 |
| 10 | 95370 | 303 | 2 | 133 | 4 | 14 | 21 | 39 | 11 | 3 | 4100 | 1.11 |
| 11 | 94685 | 257 | 2 | 130 | 4 | 15 | 26 | 42 | 13 | 3 | 4823 | 1.27 |
| 12 | 93999 | 218 | 2 | 126 | 4 | 16 | 27 | 47 | 14 | 3 | 5543 | 1.32 |
| 13 | 93313 | 185 | 2 | 123 | 4 | 16 | 27 | 51 | 14 | 4 | 6259 | 1.35 |
| 14 | 92627 | 157 | 2 | 120 | 4 | 17 | 28 | 55 | 15 | 4 | 6972 | 1.35 |
| 15 | 91943 | 134 | 2 | 117 | 4 | 17 | 28 | 58 | 15 | 4 | 7679 | 1.36 |
| 16 | 90771 | 113 | 2 | 113 | 4 | 17 | 32 | 60 | 17 | 4 | 8866 | 1.51 |
| 17 | 89612 | 95 | 2 | 110 | 4 | 17 | 33 | 62 | 17 | 5 | 10043 | 1.51 |
| 18 | 88466 | 80 | 2 | 107 | 4 | 17 | 32 | 64 | 17 | 5 | 11206 | 1.49 |
| 19 | 87332 | 68 | 2 | 104 | 4 | 17 | 32 | 65 | 16 | 5 | 12355 | 1.48 |
| 20 | 86211 | 57 | 2 | 101 | 4 | 17 | 32 | 65 | 16 | 5 | 13490 | 1.46 |
| 21 | 84128 | 47 | 2 | 97 | 4 | 17 | 39 | 66 | 19 | 5 | 15576 | 1.68 |
| 22 | 82095 | 39 | 2 | 93 | 4 | 17 | 40 | 69 | 20 | 5 | 17615 | 1.72 |
| 23 | 80110 | 33 | 2 | 90 | 4 | 17 | 39 | 73 | 20 | 5 | 19606 | 1.71 |
| 24 | 78172 | 27 | 2 | 87 | 4 | 18 | 39 | 75 | 20 | 5 | 21551 | 1.69 |
| 25 | 76280 | 23 | 2 | 84 | 4 | 17 | 38 | 77 | 20 | 5 | 23450 | 1.67 |
| 26 | 73125 | 19 | 2 | 79 | 4 | 17 | 38 | 77 | 20 | 5 | 26614 | 1.64 |
| 27 | 70101 | 15 | 2 | 75 | 4 | 17 | 37 | 77 | 19 | 5 | 29648 | 1.59 |
| 28 | 67201 | 12 | 2 | 71 | 4 | 17 | 35 | 76 | 19 | 5 | 32558 | 1.53 |
| 29 | 64421 | 10 | 2 | 68 | 3 | 16 | 34 | 75 | 18 | 5 | 35348 | 0.74 |
| Total | 2652944 | 9981 | 239 | 3386 | 112 | 417 | 795 | 1451 | 401 | 103 | 330171 | 45.29 |

A hypothetical cohort with 100,000 individuals was simulated. n the number of individuals in each Markov states

eTable 1-10 Stage cohorts and expected costs in non-screening scenario for 55-59 years

| Stage | Normal  /n | LGIN  /n | IC  /n | DFS_  IC/n | SM  /n | DFS_  SM/n | Moderate  /n | DFS_  Mod/n | Advanced  /n | PFS_  Adv/n | Dead  /n | Stage Cost/USD |
| --- | --- | --- | --- | --- | --- | --- | --- | --- | --- | --- | --- | --- |
| 0 | 99975 | 0 | 1 | 0 | 1 | 0 | 17 | 0 | 6 | 0 | 0 | 0.31 |
| 1 | 99474 | 0 | 1 | 1 | 1 | 1 | 21 | 10 | 9 | 1 | 480 | 0.83 |
| 2 | 98976 | 0 | 1 | 2 | 1 | 2 | 23 | 20 | 11 | 2 | 961 | 0.94 |
| 3 | 98481 | 0 | 1 | 3 | 1 | 3 | 24 | 29 | 13 | 3 | 1443 | 1.01 |
| 4 | 97988 | 0 | 1 | 3 | 1 | 4 | 25 | 36 | 14 | 3 | 1925 | 1.05 |
| 5 | 97497 | 0 | 1 | 4 | 2 | 4 | 25 | 42 | 14 | 4 | 2407 | 1.09 |
| 6 | 96743 | 0 | 1 | 5 | 2 | 5 | 32 | 46 | 17 | 4 | 3145 | 1.35 |
| 7 | 95994 | 0 | 1 | 6 | 2 | 6 | 34 | 54 | 19 | 5 | 3880 | 1.44 |
| 8 | 95251 | 0 | 1 | 7 | 2 | 7 | 34 | 61 | 20 | 5 | 4612 | 1.49 |
| 9 | 94514 | 0 | 1 | 8 | 2 | 7 | 35 | 66 | 20 | 5 | 5341 | 1.53 |
| 10 | 93783 | 0 | 1 | 9 | 2 | 8 | 35 | 71 | 21 | 6 | 6065 | 1.55 |
| 11 | 92555 | 0 | 2 | 10 | 3 | 8 | 42 | 74 | 23 | 6 | 7277 | 1.80 |
| 12 | 91344 | 0 | 2 | 11 | 3 | 9 | 43 | 78 | 23 | 6 | 8482 | 1.81 |
| 13 | 90149 | 0 | 2 | 12 | 3 | 10 | 43 | 81 | 23 | 6 | 9672 | 1.81 |
| 14 | 88969 | 0 | 2 | 13 | 3 | 10 | 42 | 83 | 23 | 6 | 10848 | 1.80 |
| 15 | 87805 | 0 | 2 | 14 | 3 | 10 | 42 | 85 | 23 | 6 | 12010 | 1.79 |
| 16 | 85659 | 0 | 2 | 15 | 4 | 11 | 52 | 85 | 27 | 6 | 14139 | 2.14 |
| 17 | 83565 | 0 | 2 | 16 | 4 | 12 | 54 | 91 | 28 | 7 | 16221 | 2.21 |
| 18 | 81523 | 0 | 2 | 17 | 4 | 12 | 54 | 97 | 28 | 7 | 18256 | 2.22 |
| 19 | 79530 | 0 | 2 | 18 | 4 | 13 | 53 | 101 | 28 | 8 | 20243 | 2.21 |
| 20 | 77586 | 0 | 2 | 19 | 4 | 13 | 52 | 103 | 28 | 8 | 22185 | 2.18 |
| 21 | 74359 | 0 | 2 | 20 | 4 | 14 | 51 | 104 | 28 | 8 | 25411 | 2.16 |
| 22 | 71267 | 0 | 2 | 20 | 3 | 14 | 50 | 104 | 27 | 8 | 28505 | 2.10 |
| 23 | 68303 | 0 | 2 | 20 | 3 | 14 | 48 | 103 | 26 | 7 | 31472 | 2.03 |
| 24 | 65462 | 0 | 2 | 20 | 3 | 14 | 47 | 102 | 26 | 7 | 34317 | 0.98 |
| Total | 2206753 | 0 | 40 | 273 | 65 | 211 | 977 | 1724 | 526 | 135 | 289297 | 39.83 |

A hypothetical cohort with 100,000 individuals was simulated. n the number of individuals in each Markov states

eTable 1-11 Stage cohorts and expected costs in screening without follow-up scenario for 55-59 years

| Stage | Normal  /n | LGIN  /n | IC  /n | DFS_  IC/n | SM  /n | DFS_  SM/n | Moderate  /n | DFS_  Mod/n | Advanced  /n | PFS_  Adv/n | Dead  /n | Stage Cost/USD |
| --- | --- | --- | --- | --- | --- | --- | --- | --- | --- | --- | --- | --- |
| 0 | 95481 | 4001 | 458 | 0 | 13 | 0 | 35 | 0 | 13 | 0 | 0 | 10.78 |
| 1 | 95010 | 3978 | 28 | 426 | 2 | 12 | 24 | 21 | 12 | 3 | 484 | 1.83 |
| 2 | 94542 | 3956 | 5 | 436 | 8 | 12 | 27 | 30 | 12 | 3 | 970 | 1.65 |
| 3 | 94076 | 3934 | 4 | 425 | 9 | 17 | 28 | 38 | 12 | 3 | 1455 | 1.68 |
| 4 | 93612 | 3912 | 4 | 412 | 9 | 21 | 29 | 45 | 13 | 3 | 1940 | 1.72 |
| 5 | 93151 | 3890 | 4 | 400 | 9 | 25 | 29 | 51 | 13 | 4 | 2425 | 1.74 |
| 6 | 92440 | 3857 | 4 | 387 | 9 | 28 | 35 | 56 | 16 | 4 | 3166 | 1.93 |
| 7 | 91734 | 3824 | 4 | 375 | 9 | 30 | 36 | 63 | 17 | 4 | 3904 | 2.00 |
| 8 | 91034 | 3792 | 4 | 363 | 9 | 33 | 37 | 69 | 17 | 4 | 4638 | 2.02 |
| 9 | 90339 | 3760 | 4 | 352 | 9 | 34 | 37 | 74 | 18 | 5 | 5369 | 2.04 |
| 10 | 89650 | 3728 | 4 | 341 | 9 | 35 | 37 | 78 | 18 | 5 | 6096 | 2.04 |
| 11 | 88489 | 3677 | 4 | 328 | 9 | 36 | 42 | 81 | 20 | 5 | 7309 | 2.20 |
| 12 | 87343 | 3626 | 4 | 316 | 9 | 36 | 42 | 83 | 20 | 5 | 8515 | 2.17 |
| 13 | 86212 | 3576 | 4 | 305 | 8 | 36 | 42 | 84 | 20 | 6 | 9707 | 2.14 |
| 14 | 85096 | 3526 | 3 | 294 | 8 | 36 | 41 | 85 | 20 | 5 | 10883 | 2.11 |
| 15 | 83995 | 3478 | 3 | 284 | 8 | 36 | 41 | 85 | 19 | 5 | 12045 | 2.07 |
| 16 | 81958 | 3389 | 4 | 271 | 8 | 35 | 48 | 85 | 22 | 5 | 14174 | 2.31 |
| 17 | 79970 | 3303 | 4 | 259 | 8 | 35 | 49 | 89 | 23 | 6 | 16254 | 2.33 |
| 18 | 78031 | 3219 | 4 | 247 | 8 | 35 | 49 | 93 | 23 | 6 | 18286 | 2.31 |
| 19 | 76139 | 3137 | 3 | 236 | 8 | 35 | 48 | 95 | 23 | 6 | 20270 | 2.27 |
| 20 | 74292 | 3057 | 3 | 226 | 7 | 34 | 47 | 96 | 23 | 6 | 22207 | 2.23 |
| 21 | 71217 | 2927 | 3 | 212 | 7 | 33 | 46 | 96 | 23 | 6 | 25429 | 2.18 |
| 22 | 68269 | 2803 | 3 | 199 | 7 | 32 | 45 | 95 | 22 | 6 | 28519 | 2.10 |
| 23 | 65442 | 2683 | 3 | 187 | 6 | 32 | 43 | 94 | 22 | 6 | 31482 | 2.02 |
| 24 | 62733 | 2569 | 3 | 175 | 6 | 31 | 42 | 92 | 21 | 6 | 34323 | 0.97 |
| Total | 2110253 | 87603 | 569 | 7454 | 200 | 730 | 979 | 1780 | 464 | 120 | 289849 | 58.84 |

A hypothetical cohort with 100,000 individuals was simulated. n the number of individuals in each Markov states

eTable 1-12 Stage cohorts and expected costs in screening with follow-up scenario for 55-59 years

| Stage | Normal  /n | LGIN  /n | IC  /n | DFS_  IC/n | SM  /n | DFS_  SM/n | Moderate  /n | DFS_  Mod/n | Advanced  /n | PFS_  Adv/n | Dead  /n | Stage Cost/USD |
| --- | --- | --- | --- | --- | --- | --- | --- | --- | --- | --- | --- | --- |
| 0 | 95481 | 4001 | 458 | 0 | 13 | 0 | 35 | 0 | 13 | 0 | 0 | 11.37 |
| 1 | 95581 | 3407 | 31 | 426 | 2 | 12 | 22 | 21 | 11 | 3 | 484 | 2.32 |
| 2 | 95596 | 2902 | 8 | 439 | 8 | 12 | 24 | 29 | 11 | 3 | 969 | 2.03 |
| 3 | 95539 | 2472 | 6 | 429 | 8 | 16 | 25 | 36 | 11 | 3 | 1453 | 1.99 |
| 4 | 95421 | 2105 | 6 | 419 | 9 | 21 | 26 | 42 | 11 | 3 | 1937 | 1.96 |
| 5 | 95251 | 1793 | 5 | 408 | 9 | 25 | 27 | 47 | 12 | 3 | 2421 | 1.93 |
| 6 | 94780 | 1522 | 6 | 396 | 9 | 28 | 32 | 52 | 14 | 3 | 3160 | 2.07 |
| 7 | 94273 | 1292 | 5 | 385 | 9 | 30 | 33 | 58 | 15 | 4 | 3896 | 2.09 |
| 8 | 93738 | 1097 | 5 | 375 | 9 | 32 | 34 | 63 | 16 | 4 | 4628 | 2.08 |
| 9 | 93179 | 931 | 5 | 364 | 9 | 34 | 34 | 68 | 16 | 4 | 5356 | 2.07 |
| 10 | 92601 | 790 | 5 | 353 | 9 | 35 | 34 | 71 | 16 | 4 | 6081 | 2.04 |
| 11 | 91515 | 666 | 5 | 341 | 9 | 36 | 39 | 74 | 18 | 4 | 7292 | 2.18 |
| 12 | 90425 | 562 | 4 | 330 | 9 | 37 | 39 | 76 | 18 | 5 | 8496 | 2.14 |
| 13 | 89335 | 474 | 4 | 318 | 8 | 37 | 39 | 78 | 18 | 5 | 9684 | 2.10 |
| 14 | 88246 | 400 | 4 | 308 | 8 | 36 | 38 | 79 | 18 | 5 | 10858 | 2.06 |
| 15 | 87160 | 337 | 4 | 297 | 8 | 36 | 38 | 79 | 18 | 5 | 12018 | 2.02 |
| 16 | 85095 | 281 | 4 | 283 | 8 | 36 | 45 | 79 | 20 | 5 | 14144 | 2.22 |
| 17 | 83071 | 233 | 4 | 271 | 8 | 35 | 45 | 83 | 21 | 5 | 16222 | 2.24 |
| 18 | 81090 | 194 | 4 | 259 | 8 | 35 | 45 | 86 | 21 | 6 | 18252 | 2.21 |
| 19 | 79151 | 162 | 4 | 248 | 8 | 35 | 44 | 88 | 21 | 6 | 20234 | 2.17 |
| 20 | 77255 | 134 | 3 | 237 | 7 | 34 | 44 | 89 | 21 | 6 | 22170 | 2.12 |
| 21 | 74076 | 109 | 3 | 222 | 7 | 33 | 43 | 89 | 21 | 6 | 25391 | 2.07 |
| 22 | 71025 | 89 | 3 | 208 | 7 | 33 | 41 | 88 | 20 | 6 | 28479 | 1.99 |
| 23 | 68097 | 73 | 3 | 196 | 6 | 32 | 40 | 87 | 20 | 6 | 31441 | 1.91 |
| 24 | 65289 | 59 | 3 | 184 | 6 | 31 | 38 | 85 | 19 | 5 | 34281 | 0.91 |
| Total | 2172269 | 26086 | 592 | 7697 | 200 | 730 | 904 | 1644 | 422 | 109 | 289348 | 60.30 |

A hypothetical cohort with 100,000 individuals was simulated. n the number of individuals in each Markov states

eTable 1-13 Stage cohorts and expected costs in non-screening scenario for 60-64 years

| Stage | Normal  /n | LGIN  /n | IC  /n | DFS_  IC/n | SM  /n | DFS_  SM/n | Moderate  /n | DFS_  Mod/n | Advanced  /n | PFS_  Adv/n | Dead  /n | Stage Cost/USD |
| --- | --- | --- | --- | --- | --- | --- | --- | --- | --- | --- | --- | --- |
| 0 | 99965 | 0 | 1 | 0 | 2 | 0 | 23 | 0 | 9 | 0 | 0 | 0.43 |
| 1 | 99191 | 0 | 1 | 1 | 2 | 2 | 30 | 14 | 13 | 2 | 744 | 1.15 |
| 2 | 98423 | 0 | 1 | 2 | 2 | 3 | 32 | 28 | 16 | 3 | 1489 | 1.30 |
| 3 | 97662 | 0 | 1 | 4 | 2 | 4 | 33 | 40 | 18 | 4 | 2232 | 1.40 |
| 4 | 96906 | 0 | 1 | 5 | 2 | 5 | 34 | 50 | 19 | 5 | 2973 | 1.46 |
| 5 | 96156 | 0 | 1 | 6 | 2 | 6 | 35 | 58 | 20 | 5 | 3711 | 1.51 |
| 6 | 94898 | 0 | 2 | 7 | 3 | 7 | 42 | 64 | 23 | 5 | 4950 | 1.79 |
| 7 | 93656 | 0 | 2 | 8 | 3 | 8 | 43 | 71 | 23 | 6 | 6182 | 1.82 |
| 8 | 92430 | 0 | 2 | 9 | 3 | 9 | 43 | 76 | 23 | 6 | 7399 | 1.83 |
| 9 | 91221 | 0 | 2 | 11 | 3 | 9 | 43 | 80 | 23 | 6 | 8603 | 1.84 |
| 10 | 90027 | 0 | 2 | 12 | 3 | 10 | 43 | 82 | 23 | 6 | 9792 | 1.83 |
| 11 | 87827 | 0 | 2 | 13 | 4 | 10 | 53 | 84 | 27 | 6 | 11974 | 2.20 |
| 12 | 85680 | 0 | 2 | 14 | 4 | 11 | 55 | 91 | 28 | 7 | 14108 | 2.28 |
| 13 | 83586 | 0 | 2 | 15 | 4 | 12 | 55 | 97 | 29 | 8 | 16193 | 2.29 |
| 14 | 81543 | 0 | 2 | 16 | 4 | 13 | 54 | 101 | 29 | 8 | 18230 | 2.28 |
| 15 | 79550 | 0 | 2 | 17 | 4 | 13 | 53 | 104 | 29 | 8 | 20220 | 2.26 |
| 16 | 76241 | 0 | 2 | 18 | 4 | 14 | 53 | 105 | 28 | 8 | 23527 | 2.23 |
| 17 | 73070 | 0 | 2 | 19 | 3 | 14 | 51 | 106 | 28 | 8 | 26699 | 2.18 |
| 18 | 70031 | 0 | 2 | 19 | 3 | 14 | 49 | 105 | 27 | 8 | 29741 | 2.11 |
| 19 | 67119 | 0 | 2 | 19 | 3 | 14 | 48 | 104 | 26 | 7 | 32658 | 1.02 |
| Total | 1755181 | 0 | 36 | 215 | 57 | 176 | 870 | 1460 | 461 | 116 | 241427 | 35.20 |

A hypothetical cohort with 100,000 individuals was simulated. n the number of individuals in each Markov states

eTable 1-14 Stage cohorts and expected costs in screening without follow-up scenario for 60-64 years

| Stage | Normal  /n | LGIN  /n | IC  /n | DFS_  IC/n | SM  /n | DFS_  SM/n | Moderate  /n | DFS_  Mod/n | Advanced  /n | PFS_  Adv/n | Dead  /n | Stage Cost/USD |
| --- | --- | --- | --- | --- | --- | --- | --- | --- | --- | --- | --- | --- |
| 0 | 91493 | 7530 | 861 | 0 | 25 | 0 | 66 | 0 | 25 | 0 | 0 | 14.96 |
| 1 | 90795 | 7467 | 53 | 801 | 4 | 22 | 40 | 39 | 20 | 5 | 755 | 3.26 |
| 2 | 90102 | 7404 | 10 | 817 | 15 | 21 | 44 | 53 | 20 | 5 | 1510 | 2.85 |
| 3 | 89414 | 7341 | 7 | 792 | 16 | 30 | 46 | 66 | 20 | 5 | 2262 | 2.88 |
| 4 | 88732 | 7279 | 7 | 766 | 16 | 39 | 47 | 77 | 20 | 6 | 3012 | 2.92 |
| 5 | 88055 | 7217 | 7 | 741 | 16 | 45 | 48 | 86 | 21 | 6 | 3759 | 2.94 |
| 6 | 86915 | 7118 | 7 | 713 | 16 | 51 | 53 | 93 | 23 | 6 | 5006 | 3.12 |
| 7 | 85789 | 7019 | 7 | 687 | 16 | 54 | 53 | 98 | 23 | 6 | 6248 | 3.09 |
| 8 | 84679 | 6922 | 6 | 661 | 15 | 57 | 53 | 102 | 23 | 6 | 7476 | 3.06 |
| 9 | 83582 | 6827 | 6 | 636 | 15 | 58 | 53 | 104 | 23 | 6 | 8689 | 3.02 |
| 10 | 82500 | 6733 | 6 | 613 | 15 | 59 | 52 | 106 | 23 | 6 | 9887 | 2.97 |
| 11 | 80500 | 6561 | 6 | 583 | 15 | 60 | 61 | 107 | 26 | 6 | 12076 | 3.22 |
| 12 | 78548 | 6395 | 6 | 555 | 14 | 60 | 62 | 112 | 27 | 7 | 14215 | 3.24 |
| 13 | 76643 | 6232 | 6 | 529 | 14 | 60 | 61 | 116 | 27 | 7 | 16305 | 3.19 |
| 14 | 74784 | 6073 | 6 | 504 | 13 | 60 | 60 | 119 | 27 | 7 | 18346 | 3.13 |
| 15 | 72971 | 5919 | 5 | 480 | 13 | 59 | 59 | 120 | 27 | 7 | 20340 | 3.06 |
| 16 | 69950 | 5667 | 5 | 449 | 12 | 58 | 58 | 120 | 27 | 7 | 23647 | 2.98 |
| 17 | 67054 | 5425 | 5 | 420 | 12 | 57 | 56 | 119 | 26 | 7 | 26819 | 2.86 |
| 18 | 64278 | 5194 | 5 | 393 | 11 | 55 | 54 | 117 | 25 | 7 | 29860 | 2.75 |
| 19 | 61617 | 4973 | 4 | 368 | 11 | 53 | 52 | 115 | 25 | 7 | 32775 | 1.31 |
| Total | 1608401 | 131296 | 1026 | 11508 | 281 | 959 | 1075 | 1867 | 476 | 121 | 242990 | 70.82 |

A hypothetical cohort with 100,000 individuals was simulated. n the number of individuals in each Markov states

eTable 1-15 Stage cohorts and expected costs in screening with follow-up scenario for 60-64 years

| Stage | Normal  /n | LGIN  /n | IC  /n | DFS_  IC/n | SM  /n | DFS_  SM/n | Moderate  /n | DFS_  Mod/n | Advanced  /n | PFS_  Adv/n | Dead  /n | Stage Cost/USD |
| --- | --- | --- | --- | --- | --- | --- | --- | --- | --- | --- | --- | --- |
| 0 | 91493 | 7530 | 861 | 0 | 25 | 0 | 66 | 0 | 25 | 0 | 0 | 16.09 |
| 1 | 91870 | 6392 | 60 | 801 | 4 | 22 | 35 | 39 | 18 | 5 | 755 | 4.17 |
| 2 | 92081 | 5426 | 16 | 823 | 14 | 21 | 38 | 50 | 17 | 5 | 1509 | 3.55 |
| 3 | 92152 | 4606 | 13 | 804 | 15 | 30 | 39 | 60 | 17 | 5 | 2259 | 3.42 |
| 4 | 92106 | 3909 | 12 | 783 | 16 | 38 | 41 | 69 | 17 | 5 | 3006 | 3.33 |
| 5 | 91961 | 3319 | 11 | 761 | 16 | 45 | 41 | 76 | 17 | 5 | 3749 | 3.25 |
| 6 | 91244 | 2799 | 10 | 736 | 16 | 50 | 47 | 82 | 19 | 5 | 4992 | 3.34 |
| 7 | 90462 | 2361 | 10 | 711 | 16 | 54 | 47 | 86 | 19 | 5 | 6229 | 3.25 |
| 8 | 89628 | 1992 | 9 | 687 | 15 | 56 | 47 | 90 | 19 | 5 | 7452 | 3.16 |
| 9 | 88752 | 1680 | 8 | 664 | 15 | 58 | 47 | 92 | 19 | 5 | 8660 | 3.08 |
| 10 | 87842 | 1417 | 8 | 641 | 15 | 59 | 47 | 94 | 19 | 5 | 9853 | 2.99 |
| 11 | 85914 | 1179 | 8 | 611 | 15 | 60 | 53 | 94 | 22 | 5 | 12037 | 3.17 |
| 12 | 83999 | 981 | 8 | 584 | 14 | 61 | 54 | 99 | 23 | 6 | 14172 | 3.14 |
| 13 | 82102 | 816 | 7 | 557 | 14 | 61 | 54 | 103 | 23 | 6 | 16258 | 3.07 |
| 14 | 80227 | 679 | 7 | 532 | 14 | 60 | 53 | 105 | 23 | 6 | 18294 | 2.99 |
| 15 | 78379 | 564 | 6 | 508 | 13 | 60 | 52 | 106 | 23 | 6 | 20283 | 2.90 |
| 16 | 75214 | 460 | 6 | 475 | 13 | 58 | 51 | 106 | 23 | 6 | 23587 | 2.81 |
| 17 | 72166 | 375 | 6 | 445 | 12 | 57 | 49 | 105 | 22 | 6 | 26756 | 2.69 |
| 18 | 69232 | 305 | 5 | 417 | 11 | 55 | 47 | 104 | 22 | 6 | 29795 | 2.57 |
| 19 | 66410 | 249 | 5 | 390 | 11 | 54 | 46 | 102 | 21 | 6 | 32708 | 1.21 |
| Total | 1693234 | 47037 | 1075 | 11932 | 282 | 960 | 954 | 1661 | 408 | 104 | 242353 | 74.17 |

A hypothetical cohort with 100,000 individuals was simulated. n the number of individuals in each Markov states

eTable 1-16 Stage cohorts and expected costs in non-screening scenario for 65-69years

| Stage | Normal  /n | LGIN  /n | IC  /n | DFS_  IC/n | SM  /n | DFS_  SM/n | Moderate  /n | DFS_  Mod/n | Advanced  /n | PFS_  Adv/n | Dead  /n | Stage Cost/USD |
| --- | --- | --- | --- | --- | --- | --- | --- | --- | --- | --- | --- | --- |
| 0 | 99954 | 0 | 2 | 0 | 2 | 0 | 31 | 0 | 12 | 0 | 0 | 0.56 |
| 1 | 98646 | 0 | 2 | 2 | 2 | 2 | 39 | 18 | 17 | 2 | 1270 | 1.51 |
| 2 | 97355 | 0 | 2 | 3 | 3 | 4 | 41 | 36 | 20 | 4 | 2534 | 1.64 |
| 3 | 96081 | 0 | 2 | 5 | 3 | 5 | 42 | 49 | 21 | 5 | 3787 | 1.73 |
| 4 | 94824 | 0 | 2 | 6 | 3 | 6 | 42 | 60 | 22 | 6 | 5029 | 1.78 |
| 5 | 93583 | 0 | 2 | 7 | 3 | 7 | 43 | 68 | 23 | 6 | 6259 | 1.81 |
| 6 | 91296 | 0 | 2 | 9 | 4 | 8 | 54 | 73 | 27 | 6 | 8521 | 2.22 |
| 7 | 89064 | 0 | 2 | 10 | 4 | 10 | 56 | 84 | 29 | 7 | 10735 | 2.33 |
| 8 | 86887 | 0 | 2 | 12 | 4 | 11 | 56 | 93 | 29 | 8 | 12899 | 2.36 |
| 9 | 84764 | 0 | 2 | 13 | 4 | 12 | 55 | 99 | 30 | 8 | 15014 | 2.36 |
| 10 | 82692 | 0 | 2 | 14 | 4 | 12 | 55 | 103 | 30 | 8 | 17080 | 2.34 |
| 11 | 79253 | 0 | 2 | 15 | 4 | 13 | 54 | 105 | 29 | 8 | 20516 | 2.32 |
| 12 | 75956 | 0 | 2 | 16 | 4 | 13 | 53 | 107 | 29 | 8 | 23812 | 2.27 |
| 13 | 72797 | 0 | 2 | 17 | 3 | 14 | 51 | 107 | 28 | 8 | 26973 | 2.20 |
| 14 | 69770 | 0 | 2 | 17 | 3 | 14 | 49 | 106 | 27 | 8 | 30004 | 1.06 |
| Total | 1312922 | 0 | 30 | 147 | 47 | 131 | 719 | 1108 | 371 | 91 | 184434 | 28.49 |

A hypothetical cohort with 100,000 individuals was simulated. n the number of individuals in each Markov states

eTable 1-17 Stage cohorts and expected costs in screening without scenario for 65-69years

| Stage | Normal  /n | LGIN  /n | IC  /n | DFS_  IC/n | SM  /n | DFS_  SM/n | Moderate  /n | DFS_  Mod/n | Advanced  /n | PFS_  Adv/n | Dead  /n | Stage Cost/USD |
| --- | --- | --- | --- | --- | --- | --- | --- | --- | --- | --- | --- | --- |
| 0 | 81940 | 15988 | 1829 | 0 | 52 | 0 | 139 | 0 | 52 | 0 | 0 | 25.00 |
| 1 | 80879 | 15767 | 110 | 1691 | 7 | 47 | 72 | 82 | 37 | 10 | 1298 | 6.42 |
| 2 | 79832 | 15549 | 20 | 1714 | 30 | 43 | 74 | 101 | 32 | 10 | 2594 | 5.26 |
| 3 | 78798 | 15335 | 15 | 1653 | 31 | 61 | 76 | 116 | 30 | 9 | 3876 | 5.17 |
| 4 | 77778 | 15123 | 14 | 1590 | 31 | 76 | 77 | 128 | 29 | 9 | 5145 | 5.13 |
| 5 | 76771 | 14914 | 13 | 1529 | 31 | 88 | 77 | 137 | 29 | 8 | 6401 | 5.09 |
| 6 | 74909 | 14535 | 13 | 1454 | 31 | 97 | 88 | 143 | 33 | 8 | 8688 | 5.42 |
| 7 | 73093 | 14165 | 13 | 1382 | 30 | 104 | 90 | 154 | 34 | 9 | 10924 | 5.42 |
| 8 | 71320 | 13805 | 12 | 1315 | 30 | 109 | 90 | 163 | 35 | 9 | 13112 | 5.35 |
| 9 | 69591 | 13454 | 12 | 1250 | 29 | 113 | 88 | 169 | 35 | 9 | 15249 | 5.25 |
| 10 | 67903 | 13112 | 11 | 1189 | 28 | 114 | 87 | 173 | 35 | 10 | 17338 | 5.13 |
| 11 | 65092 | 12553 | 11 | 1111 | 27 | 114 | 85 | 174 | 35 | 10 | 20788 | 4.98 |
| 12 | 62398 | 12019 | 10 | 1037 | 25 | 113 | 83 | 174 | 34 | 9 | 24097 | 4.79 |
| 13 | 59814 | 11507 | 9 | 969 | 24 | 112 | 80 | 172 | 34 | 9 | 27270 | 4.59 |
| 14 | 57338 | 11017 | 9 | 905 | 23 | 109 | 77 | 169 | 33 | 9 | 30311 | 2.19 |
| Total | 1077458 | 208844 | 2102 | 18791 | 430 | 1302 | 1283 | 2054 | 517 | 129 | 187090 | 95.20 |

A hypothetical cohort with 100,000 individuals was simulated. n the number of individuals in each Markov states

eTable 1-18 Stage cohorts and expected costs in screening with follow-up scenario for 65-69years

| Stage | Normal  /n | LGIN  /n | IC  /n | DFS_  IC/n | SM  /n | DFS_  SM/n | Moderate  /n | DFS_  Mod/n | Advanced  /n | PFS_  Adv/n | Dead  /n | Stage Cost/USD |
| --- | --- | --- | --- | --- | --- | --- | --- | --- | --- | --- | --- | --- |
| 0 | 81940 | 15988 | 1829 | 0 | 52 | 0 | 139 | 0 | 52 | 0 | 0 | 27.39 |
| 1 | 83160 | 13486 | 126 | 1691 | 7 | 47 | 61 | 82 | 33 | 10 | 1298 | 8.32 |
| 2 | 84008 | 11375 | 34 | 1729 | 29 | 43 | 61 | 94 | 26 | 9 | 2590 | 6.71 |
| 3 | 84544 | 9595 | 27 | 1681 | 31 | 60 | 62 | 104 | 23 | 8 | 3866 | 6.29 |
| 4 | 84818 | 8093 | 24 | 1628 | 31 | 75 | 63 | 111 | 22 | 7 | 5128 | 5.99 |
| 5 | 84875 | 6827 | 22 | 1575 | 31 | 87 | 64 | 117 | 21 | 6 | 6375 | 5.75 |
| 6 | 83791 | 5679 | 23 | 1504 | 31 | 96 | 72 | 121 | 24 | 6 | 8652 | 5.80 |
| 7 | 82570 | 4724 | 21 | 1439 | 30 | 104 | 73 | 128 | 25 | 6 | 10879 | 5.63 |
| 8 | 81241 | 3930 | 19 | 1376 | 30 | 109 | 73 | 135 | 26 | 7 | 13055 | 5.42 |
| 9 | 79832 | 3269 | 18 | 1314 | 29 | 112 | 72 | 139 | 26 | 7 | 15181 | 5.22 |
| 10 | 78363 | 2719 | 16 | 1255 | 28 | 114 | 71 | 142 | 26 | 7 | 17259 | 5.02 |
| 11 | 75507 | 2216 | 15 | 1176 | 27 | 114 | 70 | 143 | 26 | 7 | 20700 | 4.80 |
| 12 | 72697 | 1805 | 13 | 1101 | 26 | 114 | 68 | 143 | 26 | 7 | 24000 | 4.56 |
| 13 | 69945 | 1471 | 12 | 1031 | 24 | 112 | 66 | 141 | 25 | 7 | 27165 | 4.32 |
| 14 | 67259 | 1198 | 11 | 965 | 23 | 110 | 63 | 139 | 25 | 7 | 30199 | 1.96 |
| Total | 1194550 | 92373 | 2212 | 19464 | 430 | 1299 | 1079 | 1737 | 406 | 102 | 186348 | 103.19 |

A hypothetical cohort with 100,000 individuals was simulated. n the number of individuals in each Markov states

eTable 2 The total expected costs for different Markov states among different screening strategies (USD)

| Age | Strategy | Normal | LGIN | IC | DFS_  IC | SM | DFS_  SM | Moderate | DFS_  Mod | Advanced | PFS_  Adv | Stage Cost |
| --- | --- | --- | --- | --- | --- | --- | --- | --- | --- | --- | --- | --- |
| 40-44 | Non_scr | 0.00 | 0.00 | 0.65 | 0.23 | 1.25 | 0.32 | 22.94 | 2.58 | 11.69 | 0.36 | 40.02 |
|  | Scr_nfol | 6.02 | 0.01 | 0.65 | 0.42 | 1.02 | 0.27 | 16.38 | 1.85 | 8.28 | 0.26 | 35.14 |
|  | Scr_fol | 6.04 | 0.12 | 0.65 | 0.43 | 1.01 | 0.27 | 16.32 | 1.84 | 8.24 | 0.26 | 35.17 |
| 45-49 | Non_scr | 0.00 | 0.00 | 0.66 | 0.23 | 1.27 | 0.32 | 23.29 | 2.62 | 11.87 | 0.37 | 40.63 |
|  | Scr_nfol | 5.99 | 0.03 | 1.25 | 1.18 | 1.41 | 0.39 | 17.48 | 1.99 | 8.65 | 0.27 | 38.65 |
|  | Scr_fol | 6.07 | 0.50 | 1.26 | 1.19 | 1.41 | 0.39 | 17.22 | 1.96 | 8.52 | 0.27 | 38.78 |
| 50-54 | Non_scr | 0.00 | 0.00 | 0.66 | 0.22 | 1.27 | 0.32 | 23.26 | 2.60 | 11.83 | 0.37 | 40.52 |
|  | Scr_nfol | 5.93 | 0.09 | 2.56 | 2.66 | 2.16 | 0.62 | 19.20 | 2.20 | 9.16 | 0.29 | 44.85 |
|  | Scr_fol | 6.13 | 1.35 | 2.65 | 2.71 | 2.15 | 0.62 | 18.50 | 2.12 | 8.79 | 0.28 | 45.29 |
| 55-59 | Non_scr | 0.00 | 0.00 | 0.65 | 0.21 | 1.24 | 0.31 | 22.90 | 2.53 | 11.61 | 0.36 | 39.83 |
|  | Scr_nfol | 5.75 | 0.24 | 5.93 | 5.99 | 3.83 | 1.09 | 22.85 | 2.63 | 10.21 | 0.32 | 58.84 |
|  | Scr_fol | 6.30 | 3.52 | 6.32 | 6.18 | 3.83 | 1.09 | 21.08 | 2.43 | 9.25 | 0.29 | 60.30 |
| 60-64 | Non_scr | 0.00 | 0.00 | 0.59 | 0.17 | 1.10 | 0.26 | 20.43 | 2.15 | 10.20 | 0.31 | 35.20 |
|  | Scr_nfol | 5.51 | 0.45 | 10.41 | 9.26 | 5.34 | 1.43 | 24.92 | 2.77 | 10.40 | 0.33 | 70.82 |
|  | Scr_fol | 6.50 | 6.36 | 11.27 | 9.59 | 5.35 | 1.43 | 22.04 | 2.47 | 8.88 | 0.28 | 74.17 |
| 65-69 | Non_scr | 0.00 | 0.00 | 0.49 | 0.11 | 0.90 | 0.19 | 16.77 | 1.63 | 8.16 | 0.24 | 28.49 |
|  | Scr_nfol | 4.94 | 0.96 | 20.83 | 15.08 | 8.00 | 1.93 | 29.05 | 3.04 | 11.03 | 0.35 | 95.20 |
|  | Scr_fol | 6.86 | 12.50 | 22.72 | 15.60 | 8.00 | 1.92 | 24.17 | 2.58 | 8.56 | 0.28 | 103.19 |
